# Integrative Single-Cell and Machine Learning Analysis Develops a Glutamine Metabolism–Based Prognostic Model and Identifies MSMO1 as a Therapeutic Target in Osteosarcoma

**DOI:** 10.3390/biom15121664

**Published:** 2025-11-28

**Authors:** Hui Ma, Haiyang Zhang, Johny Bajgai, Md. Habibur Rahman, Thu Thao Pham, Chaodeng Mo, Buchan Cao, Yeong-eun Choi, Cheol-Su Kim, Kyu-Jae Lee

**Affiliations:** 1 Department of Global Medical Science, Wonju College of Medicine, Yonsei University Graduate School, Wonju 26426, Republic of Korea; mahui56@yonsei.ac.kr (H.M.); zhanghy1017@yonsei.ac.kr (H.Z.); deng@yonsei.ac.kr (C.M.); caobuchan@yonsei.ac.kr (B.C.); 2 Department of Convergence Medicine, Wonju College of Medicine, Yonsei University, Wonju 26426, Republic of Korea; johnybajgai@yonsei.ac.kr (J.B.); thao89@yonsei.ac.kr (T.T.P.); yeongeun4833@gmail.com (Y.-e.C.); 3 Department of Orthopedic Surgery, Fengjie Affiliated Hospital of Chongqing Three Gorges Medical College, Chongqing 404600, China; 4 Department of Medicine, Wonju College of Medicine, Yonsei University Graduate School, Wonju 26426, Republic of Korea

**Keywords:** osteosarcoma, glutamine metabolism, single-cell RNA sequencing, prognostic model, MSMO1, Wnt/β-catenin signaling, tumor microenvironment

## Abstract

Although metabolic pathways profoundly influence disease behavior, osteosarcoma (OS) still lacks a glutamine metabolism–based framework for patient stratification. By integrating single-cell RNA sequencing with bulk cohorts, we delineated a glutamine-associated transcriptional program and translated it into an externally validated, clinically oriented risk model. After rigorous quality control and doublet removal, 19 clusters were annotated into 10 cell types. Glutamine metabolism–related gene (GRG) scores, quantified by five orthogonal algorithms (AUCell, UCell, singscore, ssGSEA, and AddModuleScore), revealed pronounced intratumoral heterogeneity, particularly within osteoblastic cells. A composite GRG score correlated with 641 genes, defining 188 differentially expressed genes; intersecting positively correlated and up-regulated genes yielded 91 candidates. Through a 10-fold cross-validated benchmark of 10 machine-learning algorithms and 101 combinations, Step-Cox [forward] + Ridge emerged as the optimal pipeline, producing a five-gene prognostic model (GPX7, COL11A2, CPE, MSMO1, SGMS2) with moderate yet reproducible performance in independent cohorts. Functionally, stable MSMO1 knockdown in U2OS cells suppressed proliferation, migration, and invasion; increased apoptosis; altered GS, GLS, and α-ketoglutarate; and dampened Wnt/β-catenin signaling. Clinically, the model stratifies OS patients into molecular risk subgroups with distinct outcomes, supporting identification of high-risk individuals and informing personalized glutamine-targeted or combination therapies. Mechanistically, glutamine metabolism shapes the OS tumor microenvironment by modulating immune-evasion and angiogenic cues, underscoring its dual role in metabolic adaptation and immune–metabolic crosstalk. Collectively, this study establishes a single-cell–anchored, glutamine-coupled state in OS, introduces an externally validated prognostic tool with translational promise but modest discriminative power, and positions MSMO1 as a metabolic–signaling node warranting further mechanistic and in-vivo investigation.

## 1. Introduction

Primary malignant bone tumors, known as bone sarcomas, represent a rare but aggressive category of skeletal malignancies, predominantly occurring in long bones and encompassing histologically varied subtypes that confound diagnosis and treatment [1]. Among them, osteosarcoma (OS) is the most common type, noted for its aggressive local invasion, high metastatic potential, and unfavorable clinical outcomes, with a bimodal incidence peak in adolescents and individuals over 60 years of age [2,3]. Despite advancements in surgical techniques and neoadjuvant chemotherapy enhancing survival rates for patients with localized disease, the prognosis for individuals with recurrent or metastatic OS remains dismal, with five-year survival rates persistently below 30% even with aggressive multimodal therapy [4,5]. This therapeutic stagnation emphasizes the urgent necessity to elucidate the molecular and cellular mechanisms underlying treatment resistance and disease progression.

Genomic abnormalities, including mutations in TP53, RB1, and complex chromothripsis events, have been associated with OS pathogenesis; however, their translational relevance is constrained by significant interpatient variation and the absence of recurrent targetable mutations [6,7]. In recent years, cancer metabolism has emerged as a critical but still insufficiently explored area of cancer biology [8]. Among metabolic pathways, glutamine metabolism is increasingly acknowledged for its diverse functions in promoting tumor growth, maintaining redox homeostasis, and facilitating immune evasion by acting as a nitrogen and carbon donor for biosynthesis, supporting mitochondrial oxidative metabolism, and mitigating oxidative stress in metabolically reprogrammed tumor cells [9]. Despite the growing recognition of glutamine’s functional importance, the cellular and spatial heterogeneity of glutamine metabolism within the OS tumor microenvironment (TME) remains inadequately characterized. Recent functional studies have started to clarify the involvement of glutamine metabolism in the pathobiology of OS. Huang et al. revealed that the glutamine transporter SLC38A5 is overexpressed in OS, where it activates the PI3K/AKT/mTOR pathway and downstream SREBP1/SCD-1 signaling, thereby inhibiting ferroptosis and enhancing cell proliferation, migration, and invasion, which is associated with a poor prognosis [10]. Tao et al. discovered the RPS27-RPS24 fusion gene in chemoresistant OS cells, which augments GLS-mediated glutamine metabolism and mitigates copper-induced cell death, hence facilitating resistance vulnerability that can be reversed by targeting glutamine metabolism [11]. In addition to OS, Patel et al. demonstrated in a genetically modified rhabdomyosarcoma model that tumors develop a reliance on glutamine after radiation therapy, and that either genetic or pharmacological suppression of glutaminase significantly increased radiosensitivity and survival [12]. Collectively, our mechanistic insights highlight that glutamine metabolism not only facilitates OS malignancy but also represents a possible therapeutic vulnerability, reinforcing the rationale for integrating glutamine-related signatures into prognostic models and mechanism-based interventions.

The advent of deep sequencing technologies, particularly bulk RNA sequencing and single-cell RNA sequencing (scRNA-seq), has revolutionized our capacity to elucidate disease biology by facilitating high-resolution analysis of therapeutic targets and cellular heterogeneity [13,14]. In contrast to conventional bulk RNA-seq, which averages signals across diverse populations and obscures cell-specific transcriptional programs, scRNA-seq allows for the elucidation of lineage-specific trajectories and microenvironmental interactions by capturing gene expression at individual cell level [15]. Recent advancements in scRNA-seq have improved our comprehension of OS. Huang et al. applied single-cell transcriptomics to primary, recurrent, and metastatic osteosarcoma lesions and showed that LOX-high cancer-associated fibroblasts remodel the tumor immune microenvironment, promote epithelial–mesenchymal transition, and are associated with poor prognosis, highlighting CAF-targeted strategies as a promising therapeutic avenue in OS [16]. Liu et al. generated a single-cell-resolved atlas of the osteosarcoma immune microenvironment, identifying a subset of regulatory dendritic cells that recruit Tregs, reduced MHC-I expression on tumor cells, and CD24 as a novel “don’t-eat-me” signal driving immune evasion [17]. Similarly, Zou et al. demonstrated that ceramide metabolism regulates macrophage-T cell interactions, emphasizing an immune metabolic axis that affects T cell dysfunction in OS [18]. Wang et al. integrated scRNA-seq and trajectory analysis to find a subgroup with elevated lactic acid metabolism in OS, pinpointing NDUFAF6 as a biomarker mediating metabolic crosstalk with stromal and immune cells [19].

**Figure 1:**
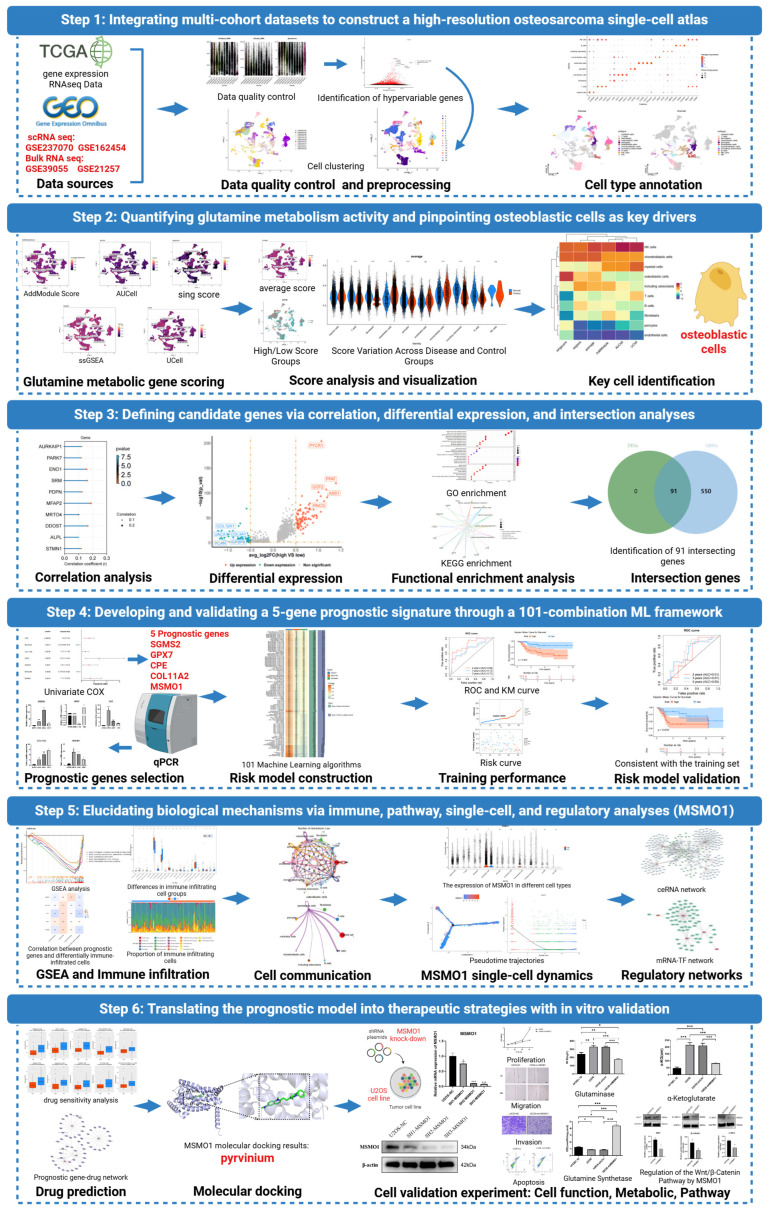
Flowchart of this research.

However, despite these discoveries, glutamine metabolism, an essential mechanism supporting cancer cell survival, redox balance, and immune evasion, remains poorly characterized at the single-cell level in OS. Considering its established associations with metastasis and therapeutic resistance across multiple malignancies, we employed integrative scRNA-seq and machine learning approaches to construct a comprehensive glutamine metabolic atlas in OS. Our objective is to delineate glutamine utilization signatures across diverse cell populations, investigate their correlations with immune modulation and tumor progression, and identify potential metabolic vulnerabilities in this highly adaptable malignancy. A detailed flowchart summarizing the conceptual framework of this study is provided in Figure 1, which presents an overview of the experimental strategy. Here, we make four key contributions. First, we integrate scRNA-seq with bulk cohorts to define a glutamine-coupled transcriptional program in osteosarcoma and quantify its intratumoral heterogeneity. Second, we develop a parsimonious five-gene prognostic model (GPX7, COL11A2, CPE, MSMO1, SGMS2) using a cross-validated machine-learning pipeline and demonstrate external validity for risk stratification. Third, we associate glutamine-metabolic states with tumor-microenvironment characteristics, uncovering transcriptional programs and immune-infiltration patterns that suggest distinct immune-regulatory and angiogenic signaling landscapes across risk groups. Fourth, we provide functional evidence that MSMO1 serves as an actionable metabolic–signaling node in vitro, where its knockdown suppresses proliferation, migration, invasion, and Wnt/β-catenin signaling while reshaping glutamine metabolism.

## 2. Materials and Methods

### 2.1. Data Source

A total of 84 OS samples, accompanied by gene expression and survival information, were retrieved from the UCSC Xena database (https://xena.ucsc.edu, accessed on 10 April 2025) and utilized as a training set. Validation cohorts were drawn from the Gene Expression Omnibus (GEO) (https://www.ncbi.nlm.nih.gov/geo/, accessed on 10 April 2025). GSE39055 (platform GPL24676) includes 37 osteosarcoma samples. GSE21257 (platform GPL10295) includes 53 samples, of which 34 developed metastasis and 19 did not within 5 years. For single-cell analyses, we merged two GEO datasets, GSE237070 (2 OS, 3 control samples) and GSE162454 (6 OS samples), yielding 8 OS and 3 control samples in total. Furthermore, 80 glutamine metabolism-related genes (GRGs) were obtained from MSigDB (http://www.broadinstitute.org/msigdb, accessed on 10 April 2025). The information of the data source shown in Table 1.

### 2.2. scRNA Analysis

To examine the distinct single-cell signatures of OS, the single-cell analysis was conducted using the “Seurat” package (version 4.1.0) [20]. Quality control was performed using the following removing thresholds: (1) cells exhibiting expression of fewer than 200 genes and genes identified in fewer than 3 cells, (2) cells with mitochondrial genes over 10%, (3) cells with the number of genes ≤200 and ≥6000, and (4) genes with count number ≤200 and ≥40,000. Then, vst method in the Find Variable Features function was employed to identify the top 2000 highly variable genes. Principal component analysis (PCA) was performed on all highly variable genes, and a total of 50 principal components (PCs) were calculated. The Scale Data function was used to scale scRNA-seq data, statistically significant PCs were determined by the Jack Straw Plot function and ElbowPlot function, and the top 30 PCs with statistical significance were finally determined for subsequent analysis. Cell clusters were identified utilizing the Find Neighbors and Find Clusters functions in “Seurat” package. For visualization, we use the RunUMAP function for nonlinear dimensionality reduction and draw the cell clustering graph based on the UMAP coordinates. Cell types were annotated by analyzing highly variable genes and published literature alongside marker genes from Cell Marker database [21,22]. Doublets among cell types were eliminated utilizing the “Doublet Finder” package (version 2.0.4) [23].

To more comprehensively and stably assess the expression levels of GRGs in various cells, we selected five algorithms to calculate GRGs scores, including AUCell, UCell, singscore, ssGSEA, and AddModuleScore. The combined application of these five algorithms helps mitigate bias [24]. AUCell and UCell were specially selected for their unique advantages in quantifying genomic activity at the single-cell level, which is crucial for accurately identifying activation patterns [25,26]. Singscore ranks the genes in a given gene set within each cell and calculates the average ranking. It provides a score based on the difference in the average rankings of positive and negative genes [27]. ssGSEA determines the enrichment of gene sets by comparing the expression values of gene sets with those of other genes and calculating the relative enrichment scores [28]. AddModuleScore calculates the weighted average expression of each cell’s gene set and normalizes these values to obtain the final score [29]. The average scores from these 5 methods were calculated, and the cell types were categorized into high and low score groups. According to the importance of cells in OS, the key cell was selected. Subsequently, Spearman analysis was conducted between all genes in key cell and GRGs scores, selecting positively linked genes based on cor > 0.1 and *p* < 0.05.

### 2.3. Identification of Prognostic Genes in OS

Differentially expressed genes (DEGs) between high and low score groups of key cells were found using the criteria |avg_log2FC| > 0.5 and *p* < 0.05. GO and Kyoto Encyclopedia of Genes and Genomes (KEGG) enrichment analyses were conducted to examine the function of up-regulated DEGs with *p* < 0.05 as the threshold. The interactions of up-regulated DEGs were further analyzed at protein levels using STRING database with a low confidence = 0.4, and the results were visualized by “Cytoscape” software (version 3.10.4) [30]. The up-regulated DEGs and positively linked genes were overlapped to derive a collection of intersection genes. Univariate Cox analysis was conducted utilizing the “survival” package (version 3.4-0) [31], with HR ≠ 1 and *p* < 0.01, followed by PH assumption test to identify prognostic genes with *p* > 0.05.

### 2.4. Construction of Prognostic Model

A total of 10 machine learning algorithms, including RSF, Enet, LASSO, Ridge, StepCox, CoxBoost, plsRcox, SuperPC, GBM, and survival-SVM, were integrated to form 101 combinations. These algorithms were evaluated using 10-fold cross-validation. The Enet, LASSO, and Ridge algorithms were executed utilizing “glmnet” package (version 4.1-6) [32], and fine-tuned in the parameter α from 0 to 1 (with an interval of 0.1) and the lambda through grid search. We employed L1 (Lasso), L2 (Ridge), and elastic net (Enet) regularization to constrain the model complexity and prevent overfitting. In addition, we also optimized the parameters of RSF, GBM and CoxBoost. Specifically, for RSF, we used mtry and nodesize to optimize the parameters. For GBM, the tuning parameters include n.trees, interaction.depth, and shrinkage. For CoxBoost, the tuning parameters were stepno and penalty. We also evaluated the feature importance through the variable importance score (RSF), relative influence (GBM), and absolute values of regression coefficients (LASSO/Enet). Step Cox was conducted via “survival” package, with direction modes configured to “both”, “backward”, and “forward”. Meanwhile, to address the issue of data imbalance, we adopted the weighted Cox model (coxph(weights=)), set the case.weights parameter in GBM and RSF, and used stratified sampling during the cross-validation process to ensure that the proportion of each compromise event was consistent.

Through area under the curve (AUC) and C-index values of model, prognostic model was subsequently developed to evaluate the risk score for OS. To assess prognostic model’s performance, receiver operating characteristic curve (ROC) curve was produced by “timeROC” package (version 1.18.0) [33]. Based on the optimal cutoff value established using the surv cutpoint function in “survminer” package (version 0.4.9) [34], the samples were categorized into high and low risk groups. Employing log-rank test, Kaplan–Meier (KM) survival curve was used to compare survival disparities between these groups (*p* < 0.05). The risk scores were ranked, and survival curve was further illustrated. Additionally, the performance of prognostic model was tested in validation sets employing identical procedures.

### 2.5. Gene Set Enrichment Analysis (GSEA)

Spearman analysis was conducted between risk score and all genes in the training set using “psych” package (version 2.2.9) [35], with correlation coefficient serving as the sorting criterion. GSEA was then performed utilizing “cluster Profiler” package (version 4.2.2) [36] with background gene set “c2.cp.kegg.v2023.1.Hs.symbols” downloaded from GSEA website (|NES| > 1, adj. *p* < 0.05).

### 2.6. Immune Infiltration Analysis

To investigate immune microenvironment of OS, “CIBERSORT” package (version 1.03) [37] was employed to evaluate immune infiltration profiles in both risk groups of training dataset, and disparities between these groups were analyzed utilizing Wilcoxon test (*p* < 0.05). Furthermore, the correlation of prognostic genes with differential immune cells was evaluated by Spearman analysis. We also used the “GSVA” package (version 1.42.0) to evaluate the abundance of 28 immune cell types in the training set (*p* < 0.05).

### 2.7. Drug Sensitivity Analysis and Prediction

IC50 value for 198 chemotherapeutics from Genomics of Drug Sensitivity in Cancer (GDSC) database was determined by “oncoPredict” package. Wilcoxon test was employed to evaluate the differences in IC50 across risk groups (*p* < 0.05). DSigDB database was utilized to uncover potential drugs with prognostic genes as targets for OS. The binding energy between key gene and corresponding drugs was subsequently assessed: 3D structure of drugs (SDF format) was retrieved from PubChem database, and transformed into PDB format using Babel GUI, whereas 3D structure of key gene was sourced from Protein Data Bank Database. Water molecules and small-molecule ligands were eliminated using PyMOL (https://www.pymol.org/), and molecular docking was conducted with Auto Dock Vina, with the outcomes shown in PyMOL.

### 2.8. Pseudotime Analysis and Cell Communication

The expression of key genes across various cell types was investigated, and its expression was contrasted across high and low-risk groups. Then, the relationship between key genes and GRGs was also assessed. Moreover, the key cell was categorized into high and low expression groups according to the expression of the key gene. The “Moncole2” package (version 2.26.0) [38] was then utilized to discern differentiation characteristics of key cells, and to analyze the expression of key genes during differentiation. The “CellPhoneDB” package (version 1.6.1) [39] was utilized for analyzing cell communication to examine connections among cell subtypes. Furthermore, GSEA was further conducted on the scRNA-seq dataset to investigate the functional roles of key genes using the KEGG background gene set consistent with above (|NES| > 1, adj. *p* < 0.05). To explore the role of key gene in other tumors such as breast cancer (BRCA) and non-small cell lung cancer (NSCLC), we analyzed the expression level of key gene in different immune cells using the TISCH database (http://tisch.compbio.cn/search-gene/, accessed on 15 November 2025). We selected the BRCA (GSE110686) and NSCLC (GSE151537) datasets respectively in the TISCH database for the expression levels analysis.

### 2.9. Cell Culture

Human normal osteoblast cells (hFOB1.19) and OS cells (U2OS, HOS, and 143B) were acquired from Immocell Biotechnology Co., Ltd. The hFOB1.19 cell line was cultivated in DF12 medium, U2OS in McCoy’s 5a, along with HOS and 143B in DMEM. Additionally, human embryonic kidney epithelial cell line HEK-293T (IM-H222) was acquired from ATCC and grown in DMEM medium. Each medium was comprised of 10% FBS and 1% P/S. All cells were cultured in a 5% CO_2_ atmosphere at 37 °C.

### 2.10. Reverse Transcription-Quantitative Real-Time PCR (RT-qPCR)

Total RNA was extracted from hFOB1.19, U2OS, HOS, and 143B using the Pure Link™ RNA Mini Kit (Thermo Fisher Scientific, Waltham, MA, USA), following the manufacturer’s instructions. All procedures were performed under RNase-free conditions. RT-qPCR was conducted using the Fast SYBR™ Green Master Mix (Thermo Fisher, Waltham, MA, USA), adhering to the manufacturer’s guidelines. Biological and technical triplicates were conducted to confirm the reliability of results. GAPDH was used to standardize the mRNA levels. Relative quantification of prognostic genes was assessed using the 2−ΔΔCt technique. Supplementary Table S1 contained specific primer sequences. Key genes were identified for further analysis based on a comprehensive analysis.

### 2.11. Western Blot

Cellular proteins were extracted on ice using RIPA lysis buffer (CST, Danvers, MA, USA) supplemented with 1% PMSF (Thermo Fisher, Waltham, MA, USA). Following SDS-PAGE separation, proteins were transferred to a PVDF membrane. Membrane was incubated with 5% nonfat milk for 30 min at ambient temperature, followed by an overnight incubation with primary antibodies (MSMO1 (SC4MOL Polyclonal Antibody, Thermo Fisher, PA5-48651, 1:1000), GAPDH Loading Control Monoclonal Antibody (Thermo Fisher, MA5-15738, 1: 10,000), WNT1 Monoclonal Antibody (Thermo Fisher, MA5-15544, 1:500), β-catenin (Beyotime, Shanghai, China; AC106, 1:1000), and c-MYC (Cell Signaling, Danvers, MA, USA; 9402S, 1:1000)) at 4 °C. Subsequently, membranes were treated with secondary antibody (goat anti-rabbit IgG (H + L) secondary antibody (Invitrogen, Carlsbad, CA, USA; 31460, 1:5000)) for 30 min at room temperature. Ultimately, target protein expression was determined using the ECL chemiluminescence technique.

### 2.12. Lentiviral Production and Transfection

A key gene lentiviral short hairpin RNA (shRNA) vector was created utilizing the pLKO.1-Puro (HG-VRA1049) vector, which cloned annealed forward and reverse oligos. Lentiviruses were produced in 293T cells using cotransfection with pLKO.1. After 48 h, the supernatant containing virus was harvested and filtered using a 0.22 µm membrane. Furthermore, OS cells were plated in a 24-well plate at 2 × 10^5^ cells per well and subsequently infected with medium containing 5 µg/mL polybrene (Merck, Darmstadt, Germany; TR-1003) and virus supernatant. The medium was replaced the following day, and cells were selected using suitable antibiotics after 48 h. Supplementary Table S2 enumerated sequences of key gene-shRNA. The expression of key genes in these cell lines was assessed using RT-qPCR and Western blot to confirm successful establishment.

### 2.13. CCK-8, Wound-Healing Assay, and Transwell Analysis

OS cells were inoculated in 96-well plates at 4 × 10^3^ cells per well. Each well received 10 µL CCK-8 solution, which was incubated for 1 h in a humidified cell incubator at 0, 24, 48, and 72 h. Absorbance at 450 nm was quantified using a microplate reader. To assess cell migration under various treatments, wound-healing assay was carried out. Cells were seeded in 6 well-plates with 6 × 10^4^ cells/well. Upon reaching 90% confluence, two perpendicular scratch lines were created utilizing a 200 µL pipette tip. The medium was then replaced with serum-free solution, and scratch widths were seen after 24 h using a 4× microscope and documented through pictures. Quantification was performed using ImageJ software (Version 1.54p).

In the transwell assay, OS cells subjected to treatment were starved for 24 h with serum-free medium. Corning^®^ Matrigel^®^ (Corning, NY, USA;) was chilled to 4 °C and combined with serum-free medium in a 1:8 ratio. Subsequently, 60 µL of the mixture was introduced into the upper chamber of the transwell, while 500 µL complete medium was added to the lower chamber. The upper chamber contained 4 × 10^4^ cells/well with various treatments. After 48 h of incubation at 37 °C in a 5% CO_2_ environment, the cells in the upper chamber were fixed with 600 µL 4% paraformaldehyde and subsequently stained with 1% crystal violet. Cells that migrated were counted utilizing an inverted light microscope, and the data was quantified using Image J software.

### 2.14. Annexin V-FITC/PI Stain

Treated cells were collected and resuspended with 1 × binding buffer at a concentration of 1 × 106 cells/mL. Cells in each group were thereafter incubated with 5 μL Annexin V-FITC for 15 min and 5 μL PI for 5 min, accompanied by gentle shaking. Subsequently, 400 μL 1 × binding buffer was introduced to each sample, and apoptotic cells were assessed by flow cytometry assay using FACSCelesta™ Fusion Flow Cytometer (BD Biosciences, San Jose, CA, USA). Data was analyzed by GraphPad Prism (version 8.0).

### 2.15. GS, GLS, and α-Ketoglutarate (α-KG) Assay

Activity of GS, along with glutaminease and α-KG levels, were quantified utilizing the respective assay kit in accordance with manufacturer’s instructions. GS (A047-1-1) and GLS (H700-1-1) assay kits were both acquired from Nanjing Jiancheng (Nanjing, China), while α-KG assay kit (S0323S) was sourced from Beyotime (Shanghai, China).

GLS level was assessed with an ELISA assay kit, adhering to manufacturer’s guidelines. In summary, 100 μL supernatant was extracted from cell culture medium following various treatments. Following the application of 300 μL washing buffer, 100 μL biological antibody solution and HRP-streptavidin were added in succession. Subsequently, 50 μL coloring solutions A and B were introduced to each well, mixed gently, and incubated for 10 to 20 min. Subsequently, 50 μL stop solution was introduced to terminate reaction, and optical density at 450 nm of each well was assessed utilizing a microplate reader.

### 2.16. Statistical Analysis

All statistical analyses for bioinformatics were conducted employing R software (version 4.2.2). In the significance analysis of various expression levels and feature values, the Wilcoxon rank-sum test or the *t*-test was employed to compare differences between two sample groups. Experimental data were expressed as mean ± standard deviation (SD). Statistical analyses were conducted using unpaired two-tailed Student’s *t*-tests or one-way ANOVA with least significant difference (LSD) post hoc tests in GraphPad Prism (version 10.0; GraphPad Software Inc., La Jolla, CA, USA). A *p* value less than 0.05 was considered statically significant.

## 3. Results

### 3.1. A Total of 10 Cell Types Were Annotated in OS

**Figure 2:**
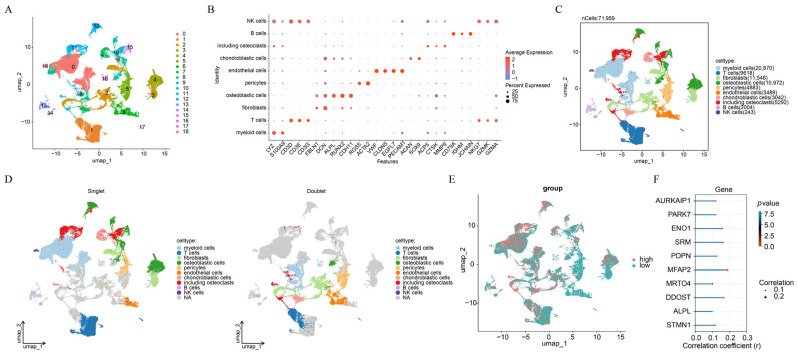
A total of 10 cell subtypes were annotated in OS. (**A**) The identification of cell clusters. (**B**) Marker genes for cell subtypes. (**C**) The annotation of cell subtypes. (**D**) The diagram of singlets and doublets. (**E**) The high and low glutamine metabolism-related genes (GRGs) score in cell subtypes. (**F**) The correlation of genes in key cell with GRGs.

To elucidate the single-cell circumstances in OS samples, scRNA-seq analysis was conducted, resulting in the remaining of 71,959 cells and 4079 genes after QC (Supplementary Figure S1A). Among the top 2000 highly variable genes and the top 30 PCs (Supplementary Figure S1B,C), 19 cell clusters were identified (Figure 2A). A total of 10 cell types were then annotated (Figure 2B,C), including myeloid cells, T cells, fibroblasts, osteoblastic cells, pericytes, endothelial cells, chondroblastic cells, osteoclasts, B cells, and natural killer (NK) cells. Following the elimination of 5397 doublets, remaining 66,562 cells had more distinct cell borders (Figure 2D). Osteoblastic cells were designated as the key cell for further examination because osteosarcoma is widely believed to arise from osteogenic-lineage progenitors rather than undifferentiated mesenchymal stem cells. Experimental and translational studies indicate that bone marrow–derived osteogenic progenitors represent a plausible cell of origin for high-grade osteosarcoma, consistent with the histological definition of malignant osteoid-producing osteoblasts as the diagnostic hallmark of this disease [40,41,42,43]. GRGs scores across cell types were computed by 5 algorithms, and all types were classified into high and low score groups according to average scores (Figure 2E). Relationship of genes in osteoblastic cells with GRGs scores was explored, yielding 641 genes as positively linked genes, such as AURKAIP1, PARK7, ENO1, SRM, PDPN, MFAP2, MRTO4, DDOST, ALPL, and STMN1 (Figure 2F).

### 3.2. CPE, COL11A2, GPX7, SGSM2, MSMO1 Were Identified as Prognostic Genes in OS

**Figure 3:**
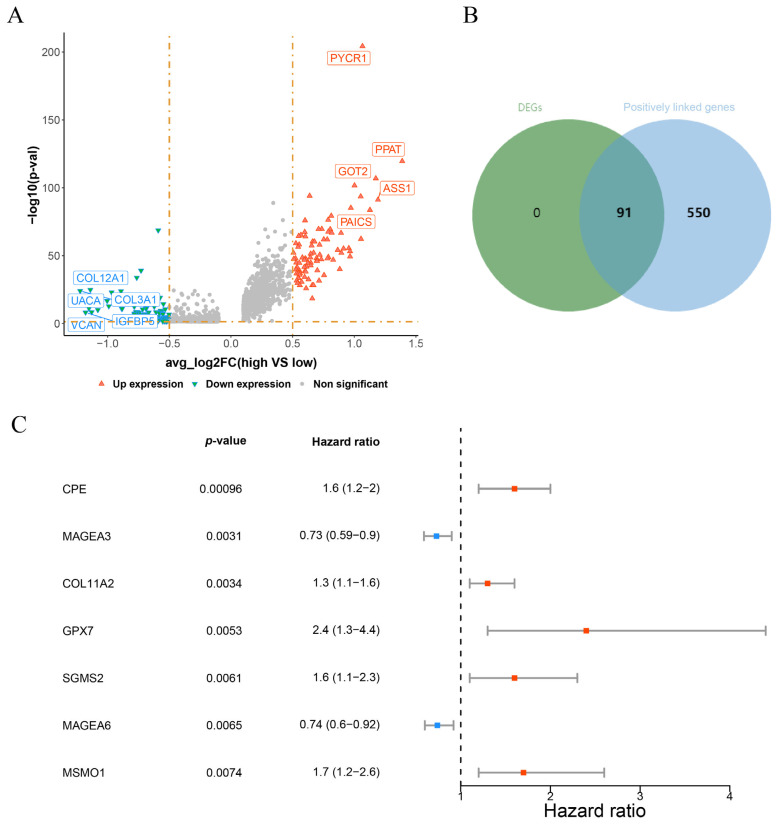
CPE, COL11A2, GPX7, SGSM2, and MSMO1 were identified as prognostic genes in OS. (**A**) The volcano plot of differentially expressed genes (DEGs). Red indicates up-regulated genes, green indicates down-regulated genes, and grey indicates genes that are not significant. (**B**) The interaction of up-regulated DEGs and positively linked genes. Green indicates up-regulated DEGs, and blue indicates positively linked genes. (**C**) The identification of prognostic genes using univariate Cox analysis. Red represents risk factors and blue represents protective factors.

A total of 188 DEGs were discovered across high and low score groups of osteoblastic cells, comprising 91 up-regulated and 97 down-regulated genes (Figure 3A). Enrichment analysis of 91 up-regulated genes identified 263 GO terms, such as glutamine metabolic process, mitochondrial matrix, and ligase activity, as well as 13 KEGG pathways: alanine, aspartate, and glutamate metabolism, biosynthesis of amino acids, and arginine biosynthesis (Supplementary Figure S2A,B). Additionally, the PPI network of up-regulated genes was constructed with 56 nodes and 123 edges (Supplementary Figure S2C). These interactions encompassed PPAT with PYCR1, PHGDH, GMPS, etc. The overlap of 91 up-regulated genes and 641 positively linked genes resulted in 91 intersection genes (Figure 3B). Univariate Cox regression analysis and PH assumption tests identified 5 prognostic genes: CPE, COL11A2, GPX7, SGSM2, MSMO1 (Figure 3C, Table 2).

### 3.3. Prognostic Model Effectively Predicts the Risk of OS

**Figure 4:**
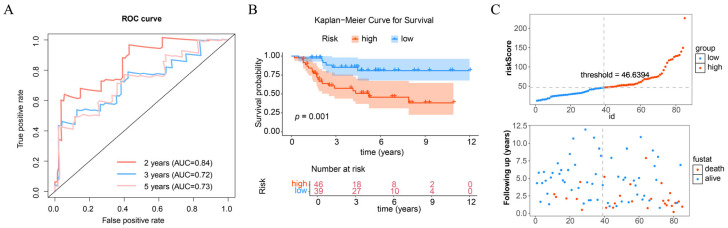
Construction of prognostic model. (**A**) Receiver operating characteristic (ROC) curve for prognostic model in the training set. (**B**) Kaplan-Meier (KM) survival curve of OS. (**C**) Survival curve for OS samples.

Utilizing 101 machine leaning combinations, the Step Cox [forward] + Ridge model attained an AUC exceeding 0.7, and a C-index value surpassing 0.6 in each dataset, establishing it as the superior model for developing the prognostic model (Supplementary Figure S3A–C). The prognostic model’s efficacy was assessed via ROC curve, yielding an AUC over 0.7 at 2, 3, and 5 years, signifying acceptable performance (Figure 4A). OS samples were categorized into high and low risk groups utilizing the optimal cutoff of the risk score. KM survival analysis indicated that low-risk samples exhibited superior survival outcomes relative to high-risk samples (Figure 4B). Furthermore, as the risk score escalated, the death rate correspondingly increased (Figure 4C). The performance of the prognostic model was additionally evaluated, with the ROC curve indicating an AUC of over 0.6 in both validation sets (Supplementary Figure S4A). Both datasets demonstrated extended survival durations in low-risk samples and increased mortality in high-risk patients (Supplementary Figure S4B,C). The results collectively indicated that the prognostic model possessed prediction capability for OS samples.

### 3.4. The Close Correlation of Immune with OS

**Figure 5:**
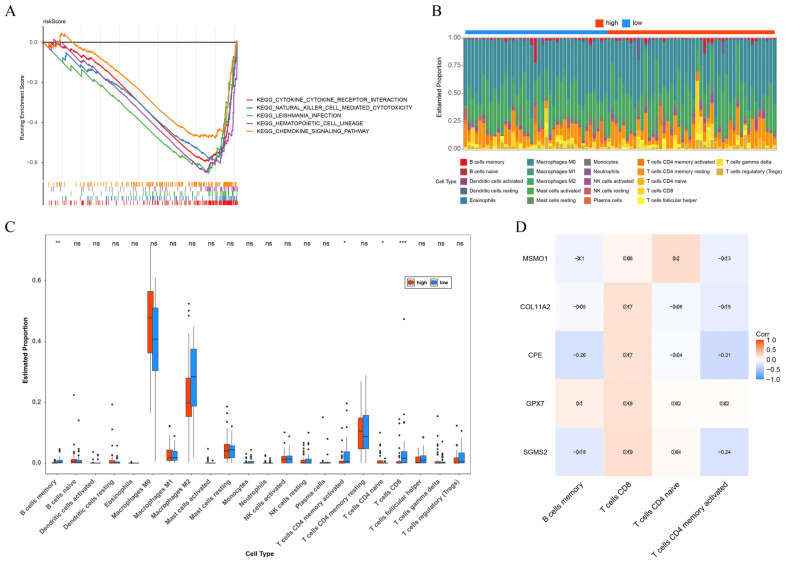
Gene set enrichment analysis (GSEA), immune infiltration analysis and regulatory network. (**A**) GSEA for risk score. (**B**) Immune infiltration profiles for OS samples in high and low risk groups. (**C**) The difference of immune cells between high and low risk groups. (**D**) The correlation between prognostic genes with differential immune cells. Blue represents negative correlation, red represents positive correlation, and those with an “×” are not significant. ns = no significance, * *p* < 0.05, ** *p* < 0.01, *** *p* < 0.001.

To deeper investigate the pathways associated with risk score, GSEA was conducted, identifying the enrichment of 26 pathways (Figure 5A). Top 5 enriched pathways-cytokine-cytokine receptor interaction, natural killer cell mediated cytotoxicity, leishmania infection, hematopoietic cell lineage, and chemokine signaling pathway-were all intricately linked to immune, highlighting the significance of immune-related mechanisms in OS. Based on this finding, immune infiltration profiles were explored in OS samples from various risk groups using the “CIBERSORT” package (Figure 5B). Marked disparities were noted in memory B cells, activated memory CD4 T cells, naive CD4 T cells, and CD8 T cells between the two risk groups (Figure 5C). Furthermore, the infiltration analysis using the “GSVA” package also indicated that there was significant difference in memory B cells between the high-risk group and the low-risk group (Supplementary Figure S5). Subsequent correlation analysis indicated that CPE had the highest negative interactions with activated memory CD4 T cells (cor = −0.31, *p* < 0.05) (Figure 5D).

### 3.5. MSMO1 Was Identified as a Key Gene in OS

**Figure 6:**
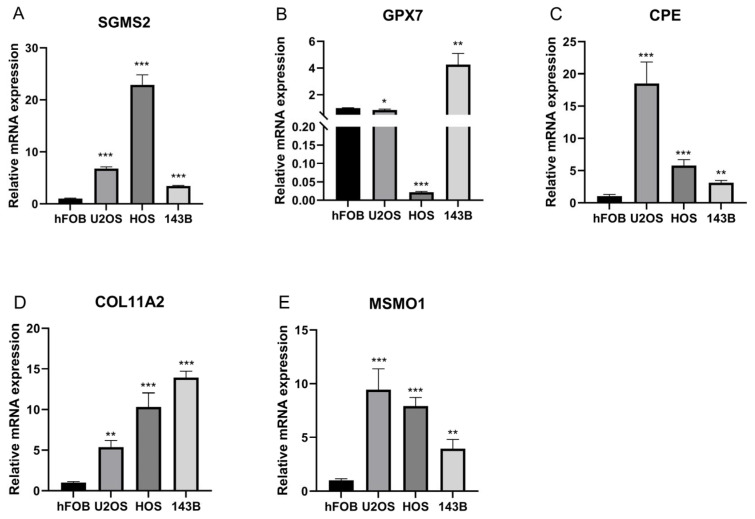
Expression of prognostic genes. (**A**–**E**) The expression of SGMS2 (**A**), GPX7 (**B**), CPE (**C**), COL11A2 (**D**), and MSMO1 (**E**). Statistical analyses were conducted using unpaired two-tailed Student’s *t*-tests and one-way ANOVA with least significant difference (LSD) post hoc tests. * *p* < 0.05, ** *p* < 0.01, *** *p* < 0.001.

The expression of prognostic genes was analyzed in human normal osteoblast cells (hFOB1.19) and OS cells (U2OS, HOS, and 143B). The finding indicated that, except for GPX7, all other genes exhibited uniformly increased expression across all OS cell types (Figure 6A–E). Due to the insufficient understanding of MSMO1’s function in OS, we identified this gene as key gene for further examination.

### 3.6. MSMO1-Mediated Regulation of the Bone Microenvironment and Potential Targeted Therapy for OS

Drug sensitivity analysis was conducted on 198 common drugs, identifying 45 drugs with notable disparities in IC50 value across two risk groups. Top 10 drugs with significance were Daporinad_1248, UMI-77_1939, Linsitinib_1510, NVP-ADW742_1932, Vorinostat_1012, Alpelisib_1560, Sabutoclax_1849, Pictilisib_1058, BI-2536_1086, KRAS (G12C) Inhibitor-12_1855 (Supplementary Figure S6A). All these drugs demonstrated elevated IC50 value in low-risk group, indicating that these drugs were sensitive to high-risk patients. Prognostic genes were utilized for medication prediction to find prospective treatments for OS. The findings indicated that 15, 6, 15, 13, and 15 drugs were respectively forecasted by CPE, COL11A2, GPX7, SGMS2, and MSMO1, and a mRNA-drug network was established with 59 nodes and 64 edges (Supplementary Figure S6B). Among these drugs, copper sulfate and trichostatin A were concurrently predicted by COL11A2, GPX7, and SGMS2. Furthermore, the molecular docking analysis was conducted between the key gene MSMO1 with its corresponding drug pyrvinium. The binding energy was −10.7 kcal/mol (Table 3), indicating the strong binding across them. Supplementary Figure S6C displayed the binding across MSMO1 and pyrvinium. Subsequently, the expression of MSMO1 was analyzed across single cell types, revealing the highest level in osteoblastic cells (Supplementary Figure S6D). A notable disparity in MSMO1 expression was seen in myeloid cells, osteoblastic cells, chondroblastic cells, and including osteoclasts across risk groups (Supplementary Figure S6E). These findings indicated that MSMO1 may influence OS progression by regulating cell interactions throughout the bone microenvironment. Correlation analysis showed a positive association between MSMO1 and the majority of GRGs, including PPAT, AADAT, and PYCR1 (Supplementary Figure S6F). To further verify the role of MSMO1 in different tumor immune microenvironments, we focused on analyzing the expression patterns of MSMO1 in immune cell subsets based on the BRCA dataset (GSE110686) and the non-small-cell lung cancer dataset (GSE151537). The results indicated that in the BRCA microenvironment, MSMO1 exhibited different expression levels in various immune cells, among which the expression of MSMO1 was the highest in Tprolif cells (Supplementary Figure S7A). In addition, MSMO1 also showed specific expression levels in different immune cells of NSCLC, and its expression level in Tprolif cells was also relatively high (Supplementary Figure S7B). This feature suggested that MSMO1 may be involved in the functional regulation of Tprolif cells, thereby influencing the activation state of the immune microenvironment. Overall, this discovery provides clues for understanding the regulatory role of MSMO1 in the tumor immune microenvironment.

**Figure 7:**
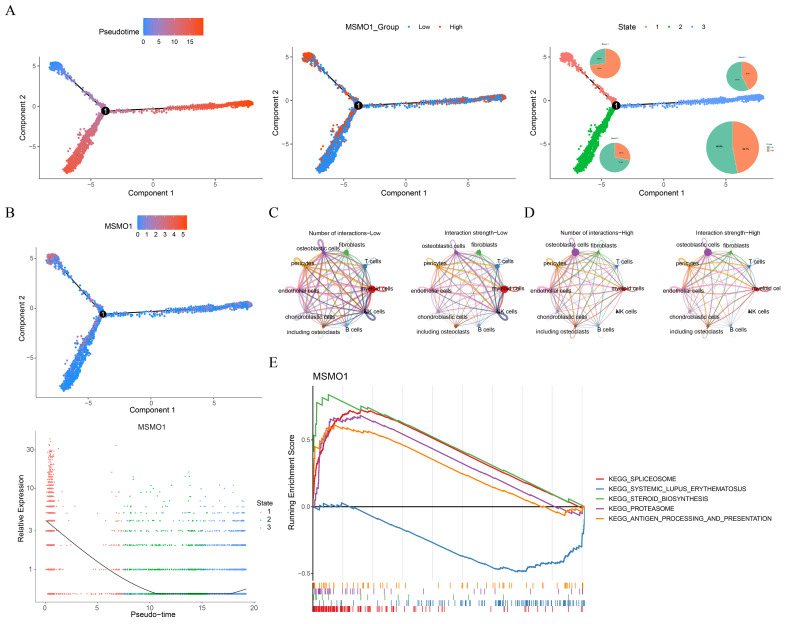
Pseudotime analysis and cell communication analysis. (**A**) Pseudotime analysis for osteoblastic cells. (**B**) The expression of MSMO1 during differentiation. (**C**,**D**) Communication among cell subtypes in low (**C**) and high-risk groups (**D**). (**E**) GSEA for MSMO1.

Pseudotime analysis of osteoblastic cells was revealed 3 stages of development. Osteoblastic cells were divided into high and low MSMO1 expression groups, with high-expression cells (53.1%) outnumbering low-expression cells (46.9%) (Figure 7A). MSMO1 expression was elevated during the initial phase of differentiation (Figure 7B). Cell communication analysis showed that osteoblastic cells in low-risk groups exhibited increased connections with other cell subtypes (Figure 7C,D). GSEA of MSMO1 in single cell dataset showed enrichment in 48 KEGG pathways. Top 5 enriched pathways comprised the spliceosome, steroid biosynthesis, proteasome, and antigen processing and presentation (Figure 7E). These pathways were intricately linked to tumor activities including proliferation, immune evasion, drug resistance, and metabolic reprogramming. This confirmed the function of MSMO1 in the advancement of OS.

### 3.7. Knock Down of MSMO1 Inhibited the Activity of U2OS Cells

**Figure 8:**
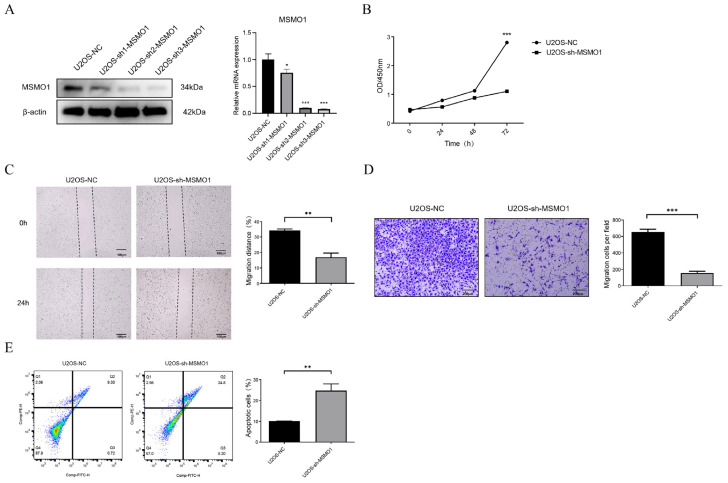
Knock down of MSMO1 inhibited the activity of U2OS cells. (**A**) The expression of MSMO1 in OS cells with sh-MSMO1. (**B**–**D**) The influence of MSMO1 in cell proliferation (**B**), migration (**C**), and invasion (**D**). (**E**) The correlation of apoptosis rate with MSMO1. β-catenin served as loading control. Data are means ± SD and were analyzed by two-tailed Student’s *t*-test, *n* = 3. The original Western blot figure is in the Supplementary File S2. * *p* < 0.05, ** *p* < 0.01, *** *p* < 0.001.

Furthermore, the expression of MSMO1 was the highest in U2OS cells and had significance, hence, U2OS cells expressing sh-MSMO1 were created. RT-qPCR and Western blot demonstrated a marked decrease in MSMO1 gene expression in U2OS cells with sh-MSMO1 compared to control (Figure 8A), confirming the successful development of the sh-MSMO1 U2OS cell model. To investigate the function of MSMO1 in U2OS cells, CCK-8, wound healing, and transwell experiments were performed. The findings indicated that MSMO1 knockdown suppressed the proliferation, migration, and invasion (Figure 8B–D), implying that MSMO1 may serve as a crucial factor in OS advancement. Moreover, apoptosis analysis demonstrated an elevated apoptosis rate in U2OS cells with sh-MSMO1 (Figure 8E), hence reinforcing the putative function of MSMO1 knockdown in facilitating OS cell survival.

### 3.8. MSMO1 Regulated Glutamine Metabolism via Wnt/β-Catenin Pathway

**Figure 9:**
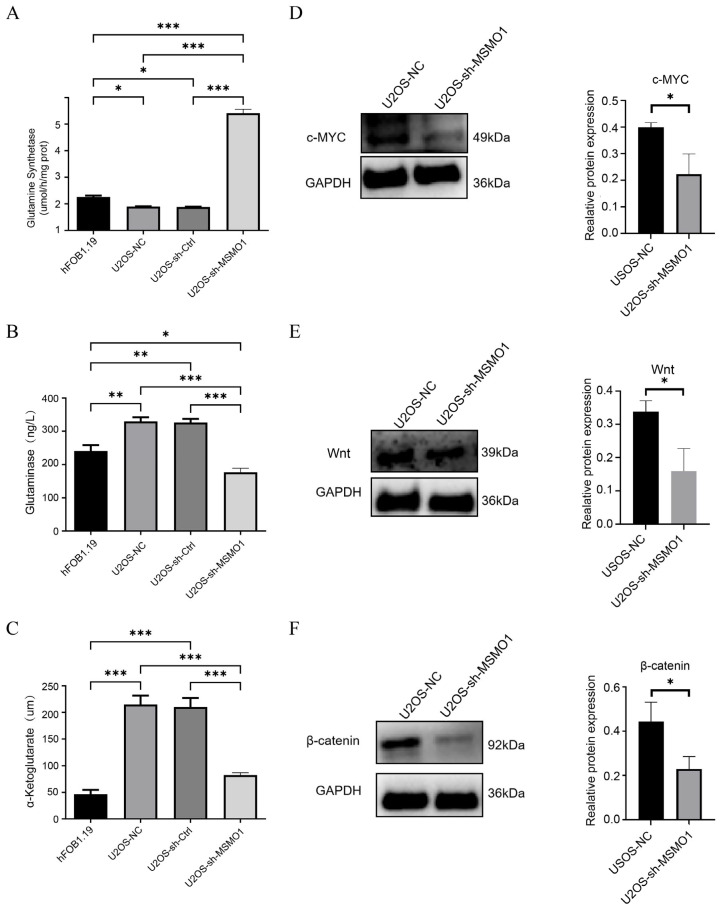
MSMO1 regulated glutamine metabolism via Wnt/β-catenin pathway. (**A**–**C**) The influence of MSMO1 in glutamine synthetase (**A**), glutaminase (**B**), and α-Ketoglutarate (**C**). (**D**–**F**) The expression of c-MYC (**D**), Wnt (**E**), and β-catenin (**F**). GAPDH served as loading control. The different shades in the bar chart represent different experimental groups and treatment conditions, including human normal osteoblasts cells (hFOB1.19) and OS cells (U2OS), as well as the U2OS control group and the U2OS cell group expressing sh-MSMO1. Statistical analyses were conducted using unpaired two-tailed Student’s *t*-tests and one-way ANOVA with least significant difference (LSD) post hoc tests, * *p* < 0.05, ** *p* < 0.01, *** *p* < 0.001.

Metabolic disorders constitute a significant characteristic of malignancies. Rapidly multiplying cancer cells predominantly depend on glutamine for their survival and proliferation [44]. To determine the influence of MSMO1 in glutamine metabolism, GS activity, GLS level and α-KG level were measured in hFOB1.19 and U2OS cells. Findings indicated that, in contrast to hFOB1.19, U2OS cells demonstrated decreased GS activity, alongside elevated GLS and α-KG levels. Furthermore, U2OS cells expressing sh-MSMO1 demonstrated increased GC activity, but GLS and α-KG levels were reduced (Figure 9A–C). The data indicated that MSMO1 may facilitate cell proliferation and metabolism by suppressing endogenous synthesis and augmenting glutamine degradation. C-MYC, a principle transcriptional activator of GLS, is recognized for its direct regulation of GLS expression and function [45]. Consequently, we assessed the level of c-MYC in U2OS cells, which were observed to be decreased in cells with sh-MSMO1 (Figure 9D). Given that c-MYC is a pivotal component of the Wnt/β-catenin pathway, we evaluated expression of Wnt and β-catenin as well. The findings demonstrated a uniform reduction in both Wnt and β-catenin expression in U2OS cells with sh-MSMO1 (Figure 9E,F). These results indicated that MSMO1 regulated glutamine metabolism in OS through the Wnt/β-catenin pathway.

## 4. Discussion

OS is the most prevalent primary malignant bone tumor, and it is characterized by significant aggressiveness and unfavorable prognosis. Despite progress in surgical, chemotherapeutic, and radiotherapeutic interventions, the 5-year survival rate, especially for recurrent or metastatic conditions (with a long-term survival rate under 20%), has not significantly improved, highlighting the necessity for novel mechanistic insights and targetable weaknesses [46]. The scRNA-seq analysis revealed that osteoblastic tumor cells exhibited a high-glutamine state, aligning with a model in which glutamine facilitates tricarboxylic-acid (TCA) anaplerosis, supports nucleotide/amino-acid biosynthesis, and sustains redox balance to promote proliferation and motility in OS. These conclusions correspond with established cancer metabolism research indicating that glutamine uptake and catabolism (via GLS and subsequent dehydrogenases/transaminases) supply α-KG to the TCA cycle and furnish precursors and reducing equivalents that mitigate oxidative stress during accelerated growth [9].

Additionally, the integration of single-cell and bulk transcriptomes revealed 5 prognostic genes linked to glutamine metabolism-CPE, COL11A2, GPX7, SGSM2, and MSMO1-that demonstrated significant risk stratification for OS samples. CPE has been associated with tumor cell migration, invasion, and unfavorable prognosis in several malignancies, and it was recently identified as a prognostic marker co-expressed with osteoblastic genes in OS [47], consistent with our incorporation of CPE in the risk model. COL11A2, modulates fibrillogenesis, constrains fibril diameter and spacing, and consequently influences matrix mechanics with indirect implications for mechano transduction. COL11A2 is essential for skeletal morphogenesis [48], and has been documented to be up-regulated in several cancers [49]. These findings were consistent with our integrated OS analysis, wherein COL11A2 was situated in the “positively correlated and up-regulated” core, correlated with the composite GRGs score in osteoblastic cells, and was included in the externally validated 5 gene prognostic signatures. GPX7, an ER-resident oxidative stress sensor of the GPX family, alleviates oxidative and proteotoxic stress by interacting with the PDI/GRP78 folding machinery, consistent with the increased anabolic and redox demand driven by glutamine-fueled growth [50]. SGMS2 catalyzes the transfer of phosphocholine from phosphatidylcholine to ceramide, producing sphingomyelin and diacylglycerol, hence altering ceramide/sphingomyelin homeostasis, and structuring lipid-raft platforms [51]. In breast cancer, SGMS2 overexpression increases TGF-β1 level and activates TGF-β/Smad signaling, thereby inhibiting apoptosis and promoting invasiveness [52]. Additionally, germline SGMS2 variants are associated with early-onset osteoporosis and skeletal dysplasia with bone fragility, highlighting the role of plasma-membrane sphingomyelin metabolism in osteoblast/osteocyte biology [53].

MSMO1, a typical target of cholesterol biosynthesis, has been discovered to be up-regulated in correlation with drug resistance, tumor cell proliferation, and suppression of apoptosis in several cancers [54,55]. Given the limited understanding of MSMO1 in osteosarcoma, we prioritized this gene for mechanistic interrogation. In our study, MSMO1 knockdown in U2OS cells consistently reduced proliferation, migration, and invasion while increasing apoptosis, indicating a functional contribution to OS cell aggressiveness. At the metabolic level, MSMO1 silencing enhanced GS activity, decreased GLS abundance, and lowered intracellular α-ketoglutarate, collectively suggesting that MSMO1 influences glutamine utilization and anaplerotic flux. Concomitantly, MSMO1 depletion attenuated β-catenin and c-MYC, in line with the established role of c-MYC as a transcriptional activator of GLS [56].

Taken together, these findings support a model in which MSMO1 shapes osteosarcoma behavior through coordinated regulation of sterol metabolism, Wnt/β-catenin–c-MYC signaling, and glutamine catabolism. Mechanistically, MSMO1-driven sterol remodeling likely alters membrane cholesterol and sphingolipid composition, affecting receptor-enriched microdomains known to potentiate Wnt/β-catenin signaling, a pathway broadly recognized for promoting OS proliferation and immune evasion. This hierarchical organization aligns with the established model in which MYC operates as a central oncogenic hub coordinating transcription, metabolism, and biosynthetic programs [57].Our observation that MSMO1 silencing suppresses β-catenin and c-MYC aligns with this sterol-dependent regulatory axis and provides a plausible link between membrane lipid organization and glutamine metabolic reprogramming [58,59]. The downstream metabolic effects are consistent with prior reports showing that GLS1 activity fuels tumor growth and metastatic progression in OS, and that genetic or pharmacologic inhibition of GLS1 reduces migratory and invasive potential [60]. Similarly, the documented dependence of OS cells on the glutamine transporter SLC1A5 (ASCT2) further underscores their reliance on exogenous glutamine to sustain anabolic and redox balance. Altogether, this constellation of evidence positions MSMO1 as a mechanistically anchored, metabolically connected regulator of OS progression-linking sterol homeostasis, Wnt pathway activation, and glutamine-dependent metabolic fitness.

Beyond intrinsic metabolism, our research demonstrates that glutamine pathways interface with the immune microenvironment. Bulk deconvolution demonstrated notable disparities in immune cell populations between OS and control, as well as their associations with prognostic genes, such as CPE, which correlated with activated memory CD4 T cells (cor = −0.31, *p* < 0.05), linked our glutamine-coupled program to immunological contexture. These observations correspond with recent OS single-cell/spatial investigations that define intricate immune-stromal niches and immunoregulatory conditions in primary and metastatic lesions [61]. High-glutamine tumor environment can diminish extracellular glutamine and alter nutrient gradients, consequently limiting T-cell effector function and promoting suppressive myeloid programs. In contrast, inhibition of the glutamine pathway can reprogram myeloid cells and augment antitumor immunity in preclinical settings. These concepts correspond with our immune-deconvolution signals that connect the risk program to immunological contexture [62,63]. Notably, the correlation between metabolic condition and immunological contexture has direct implications for OS, a disease whose immune-modulatory status has not consistently provided benefits. Nutrient competition and glutamine flux mechanistically shape both tumor fitness and antitumor immunity. Targeting this axis can influence redox balance, anaplerosis, and biosynthesis in cancer cells. While variably affecting T-cell function, thus requiring biomarker-guided application instead of a uniform approach. Concurrently, canonical Wnt/β-catenin signaling is a well-characterized mediator of T-cell exclusion [64]; our in vitro studies indicated that MSMO1 silencing attenuates β-catenin/c-MYC, suggesting a pathway to relieve a non-redundant barrier to immune infiltration in glutamine-dependent osteoblastic states. Collectively, this evidence supports two immediate clinical applications: (i) risk stratification and trial enrichment using the prognostic gene score to nominate patients for glutamine-axis interventions and metabolism-immunity combinations; and (ii) development of MSMO1-informed strategies, either directly or through sterol/lipid-raft modulation, as testable mechanisms to integrate metabolic regulation with Wnt pathway suppression in OS. We found through two calculation methods (“CIBERSORT” package and “GSVA” package) that the infiltration level of memory B cells were relatively high in the low-risk group. Studies have shown that inhibiting memory B cells can promote the occurrence and development of OS [65]. This indicated that memory B cells have important immunomodulatory functions in OS.

In OS, where survival improvements have stagnated despite advancements in MAP-based chemotherapy and surgical advances, biologically anchored risk models are increasingly recognized as necessary for guiding rational trial design [66]. Our glutamine metabolism-related prognostic genes aid this initiative by delivering a reproducible, clinically applicable assessment of metabolic activity that stratifies diagnosed individuals. Recent single-cell studies have commenced the reformation of OS categorization and prognosis. Cheng et al. leveraged single-cell RNA sequencing to demonstrate that osteosarcoma lesions are highly infiltrated by metabolically active regulatory T cells, mapped their CXCL-mediated interactions with osteoblastic, endothelial, and myeloid cells, and derived a Treg-based prognostic model that also predicted sensitivity to multi-kinase inhibitors such as sunitinib, sorafenib, and axitinib [67]. Building on these observations, Zhang et al. established three molecular subgroups based on CSC differentiation trajectories, which were validated in 138 clinical specimens, and linked to both prognosis and therapeutic sensitivity [68]. In addition, Truong et al. created the first human mesenchymal differentiation atlas using a tissue-engineered model and scRNA-seq, showing that stem-like transcriptional phenotypes in OS correlate with inferior survival [69]. Zeng et al. concurrently identified chemoresistant stem-like clusters by single-cell profiling and derived a ten-gene risk score that markedly outperformed bulk DEG-based classifiers in independent cohorts (AUCscRNA = 0.84 vs AUCbulk = 0.54) [70]. Together, these models underscore that integrating single-cell resolution with bulk validation enables extraction of signatures that are both biologically grounded and potentially actionable. Relative to recently proposed osteosarcoma classifiers based on differentiation trajectories or stem-like transcriptional programs, our model captures a complementary metabolic axis rather than developmental states or bulk DEG patterns. By explicitly incorporating glutamine dependence, immune contexture, and MSMO1-centered signaling, the glutamine-based signature provides a mechanistically anchored framework for risk stratification. Nonetheless, as with existing OS prognostic tools, the AUC and C-index of our model remain modest, highlighting that prospective validation and multi-omics refinement will be required before clinical implementation. Against this backdrop, our glutamine-based classifier captures a distinct metabolic vulnerability and offers a realistic instrument for biomarker-enriched enrollment into metabolism-focused trials. The findings from the CANTATA study in renal cell carcinoma, where glutaminase inhibition was ineffective in an unselected cohort despite a robust mechanistic rationale [71], underscores that success in OS will depend on integrating precise molecular stratification with judicious therapeutic combinations to avert metabolic compensation.

Our analyses initially combined scRNA-seq and machine learning to elucidate prognostic genes linked to glutamine metabolism, which shown efficacy in predicting overall survival risk of OS. Subsequent experiment data indicated that targeting MSMO1-regulated glutamine metabolism may be the valuable pathway for the therapy of OS. However, there are several limitations in this study. This work integrates retrospective single-cell and bulk cohorts with inevitable platform and annotation heterogeneity; despite stringent QC and harmonization, residual batch effects may remain. Moreover, the sample size of the validation set (GSE39055 and GSE21257) was relatively limited, which may pose a risk of overfitting. In the future, we will further enhance the applicability of the prognostic model by integrating multi-center, large-sample prospective cohorts. The “glutamine activity” state was inferred from transcriptomic signatures (five algorithms) and supported by GS/GLS and α-KG readouts, but we did not perform isotope-tracing metabolomics or lipidomics to quantify flux or sterol composition. The immune infiltration analysis in this study mainly relies on algorithmic estimation and still lacks direct evidence at the experimental level. It will be further confirmed through flow cytometry or Immunohistochemistry in the future. In this study, through 101 combinations of machine learning algorithms, the Step Cox [forward] + Ridge model was screened as an excellent model (AUC > 0.7, C-index > 0.6) for developing prognostic models. However, it has to be admitted that its predictive effect was still at a medium level. In future research, we will expand the sample size and further optimize the model construction, with the aim of providing more accurate and reliable prognostic assessment tools for OS. The prognostic gene risk model was externally tested only in retrospective datasets; prospective calibration, assessment of independence from clinical covariates, and pre-specified cut points are needed. Functional studies focused on MSMO1 in one line (U2OS) using shRNA, without rescue/overexpression, CRISPR confirmation, Wnt reporter assays, or in-vivo models. These gaps define next steps: prospective, multi-center validation; flux and lipidomic profiling; multi-model gain/loss-of-function with rescue and reporters; and spatial/TME-aware and in-vivo evaluation.

## 5. Conclusions

Integrating single-cell and bulk transcriptomes, we delineate a single-cell–anchored, glutamine-coupled program in OS, preferentially expressed by osteoblastic tumor cells, and distill it into a five-gene signature (GPX7, COL11A2, CPE, MSMO1, SGMS2) with moderate yet reproducible prognostic performance across independent cohorts. Stable shRNA silencing of MSMO1 in U2OS cells curtailed proliferation, migration, and invasion, increased apoptosis, shifted the GS/GLS–α-ketoglutarate axis, and attenuated β-catenin/c-MYC signaling, supporting MSMO1 as a putative metabolic–signaling regulator rather than a fully validated therapeutic target. Together, these findings provide a hypothesis-generating stratification framework and a mechanistic foothold that warrants further multi-omics refinement, rescue/overexpression validation, and prospective evaluation before clinical translation.

## Figures and Tables

**Table 1:** The information of the data source.

Use in Study	Accession	Source	Samples Size (*n*)	Note
Training set	TARGET-OS	UCSC Xena	84	Overall survival time/status, age, sex, et al.
Validation set	GSE39055	GEO	37	37 surgical resection specimens
	GSE21257	GEO	53	34 metastatic/19 non-metastatic
Sc RNA seq	GSE237070	GEO	5	2 OS and 3 control samples
	GSE162454	GEO	6	6 OS samples
GRG set	GRGs	MSigDB	80 genes	glutamine metabolism-related genes

**Table 2:** The results of proportional hazards (PH) assumption test.

ID	*p* Value
CPE	0.222603810060145
MAGEA3	0.559377399103922
COL11A2	0.0860725731817447
GPX7	0.510907341076549
SGMS2	0.47011708773789
MAGEA6	0.470480197168624
MSMO1	0.269433637199621

**Table 3:** The molecular docking of MSMO1 and pyrvinium.

Gene	PDB	Chemical Name	kcal/mol
MSMO1	Q15800	pyrvinium	−10.7

## Data Availability

All the scRNA and bulk RNA sequencing data of the osteosarcoma that is used in this research are publicly available. The original contributions presented in this study are included in the article/Supplementary File S2.

## Supplementary Materials

The following supporting information can be downloaded at: https://www.mdpi.com/article/10.3390/biom15121664/s1, Supplementary Figure S1. Single-cell RNA sequencing (scRNA-seq) analysis. Supplementary Figure S2. Pathway enrichment analyses and Protein-protein interaction (PPI) network analysis of 91 upregulated differentially expressed genes (DEGs). Supplementary Figure S3. Selection of the optimal model. Supplementary Figure S4. Validation of the prognostic model. Supplementary Figure S5. Employing the “GSVA” package to evaluate differences in immune cell infiltration levels between high- and low-risk groups of osteosarcomas. Supplementary Figure S6. Potential targeted therapy for OS. Supplementary Figure S7. The expression levels of MSMO1 in different immune cells of breast cancer (BRCA) and non-small cell lung cancer (NSCLC). Supplementary Table S1. The sequences of primers. Supplementary Table S2. The sequences of primers for sh-MSMO1. Supplementary File S2: Original Western blot images of Figures 8 and 9.

## Abbreviations

The following abbreviations are used in this manuscript:

OS	Osteosarcoma
scRNA seq	Single cell RNA sequencing
GS	Glutamine synthetase
GLS	Glutaminase
TME	Tumor microenvironment
GEO	Gene expression omnibus
GRGs	Glutamine metabolism-related genes
DEGs	Differentially expressed genes
KEGG	Kyoto encyclopedia of genes and genomes
GSEA	Gene set enrichment analysis
GDSC	Genomics of drug sensitivity in cancer
shRNA	short hairpin RNA
α-KG	α-ketoglutarate
MSMO1	Methylsterol monooxygenase 1
GPX7	Glutathione peroxidase 7
COL11A2	Collagen type XI alpha 2 chain
CPE	Carboxypeptidase E
SGMS2	Sphingomyelin synthase 2
PI3K	Phosphatidylinositol-3 kinase
AKT	AKT serine/Threonine kinase
mTOR	Mechanistic target of rapamycin
PCs	Principal components
PCA	Principal component analysis
STRING	Search tool for the retrieval of interacting genes/proteins
PDB	Protein data bank
GAPDH	Glyceraldehyde-3-phosphate dehydrogenase
RIPA	Radioimmunoprecipitation assay
PMSF	Phenylmethylsulfonyl fluoride
PVDF	Polyvinylidene difluoride
SD	Standard deviation
