# Supplementary material for: Integrative Single-Cell and Machine Learning Analysis Develops a Glutamine Metabolism–Based Prognostic Model and Identifies MSMO1 as a Therapeutic Target in Osteosarcoma
Source: Biomolecules. 2025 Nov 28;15(12):1664. doi: 10.3390/biom15121664 (PMC12731238; doi:10.3390/biom15121664)
Supplement: Supplementary file 1 [file biomolecules-15-01664-s001.zip › Experimantal original images of Figure 8 and 9.pptx]

## Slide 1
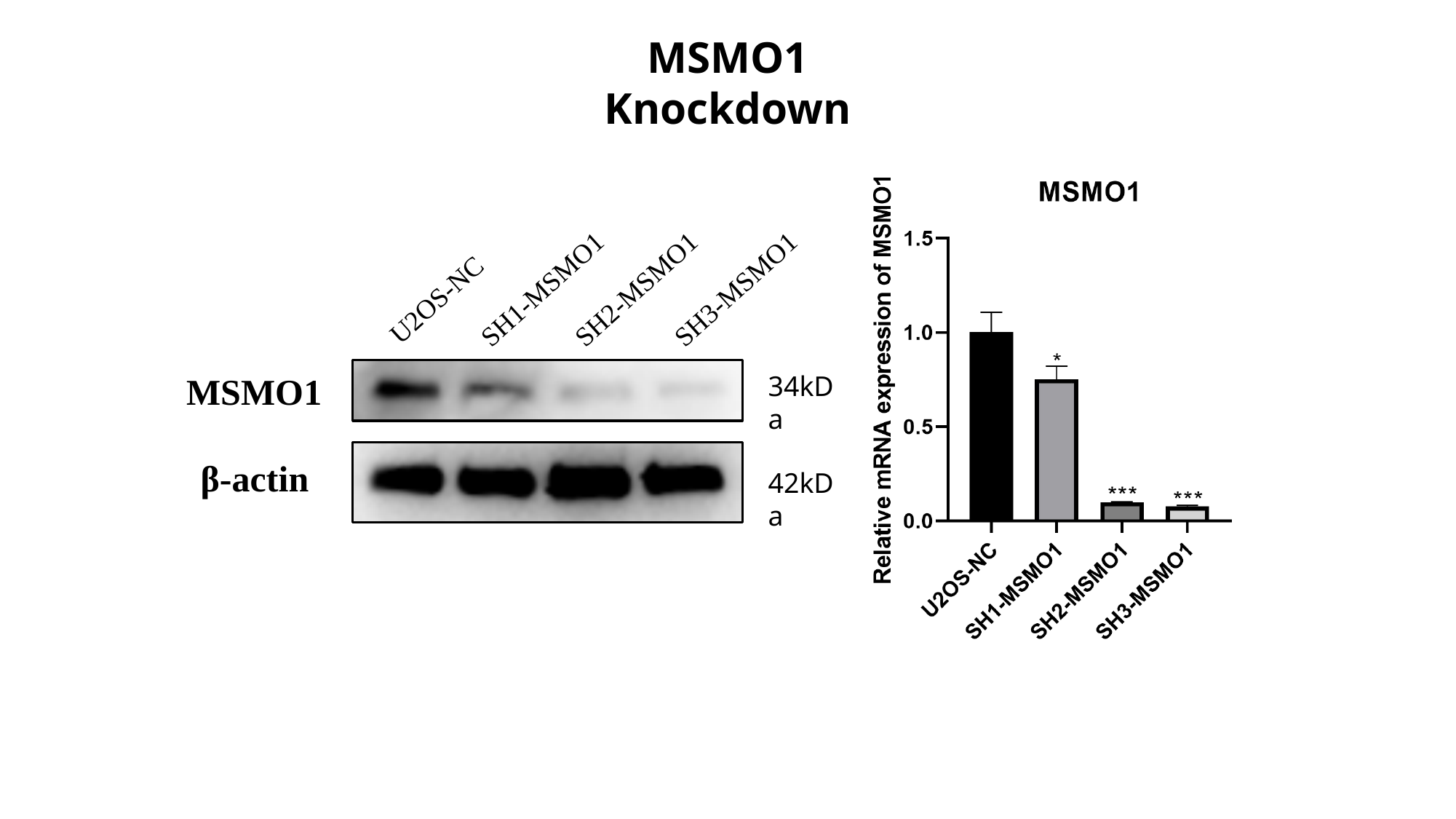

MSMO1 Knockdown
U2OS-NC
SH1-MSMO1
SH2-MSMO1
SH3-MSMO1
MSMO1
34kDa
β-actin
42kDa

## Slide 2
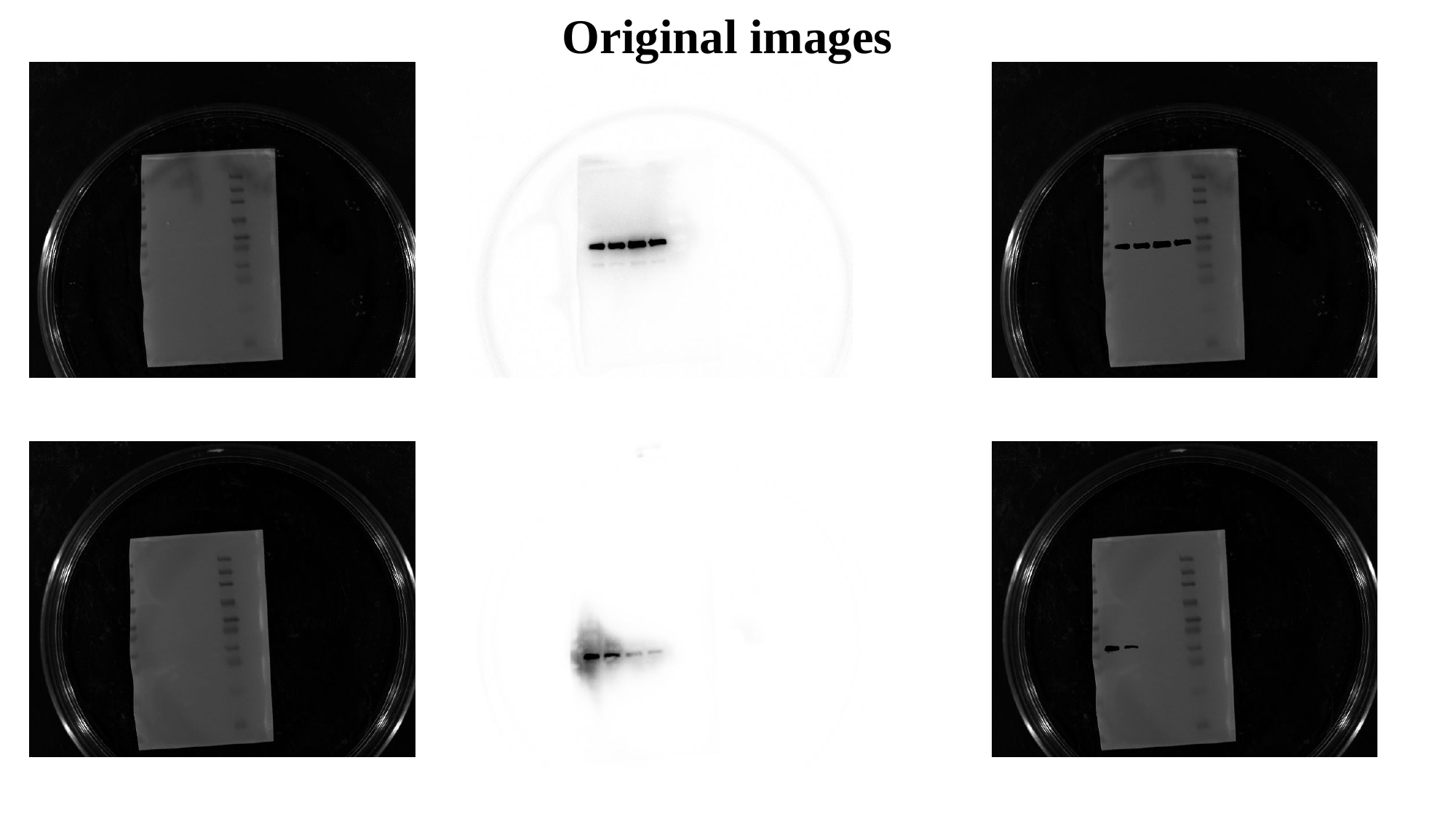

Original images

## Slide 3
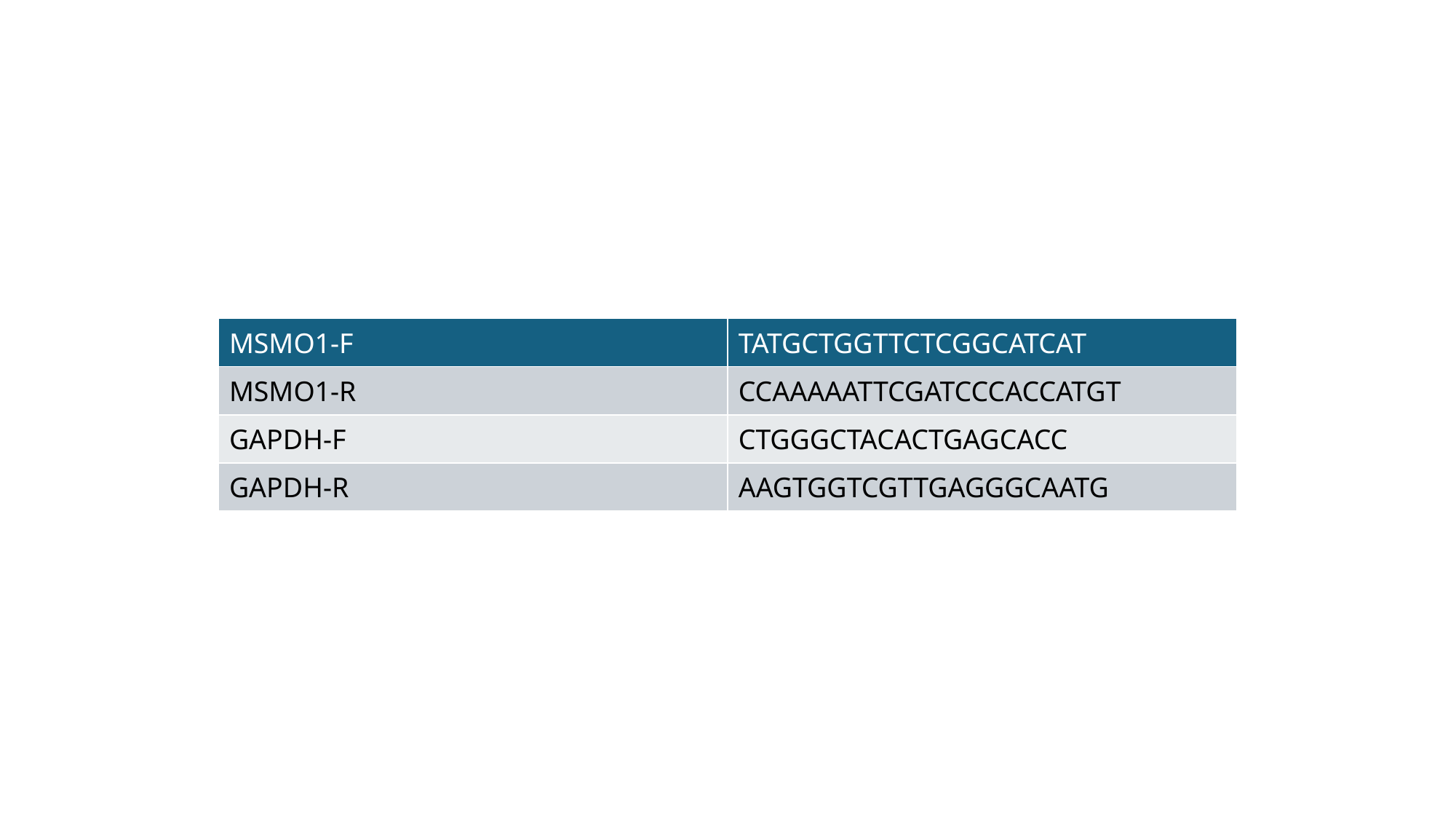

| MSMO1-F | TATGCTGGTTCTCGGCATCAT |
| --- | --- |
| MSMO1-R | CCAAAAATTCGATCCCACCATGT |
| GAPDH-F | CTGGGCTACACTGAGCACC |
| GAPDH-R | AAGTGGTCGTTGAGGGCAATG |

## Slide 4
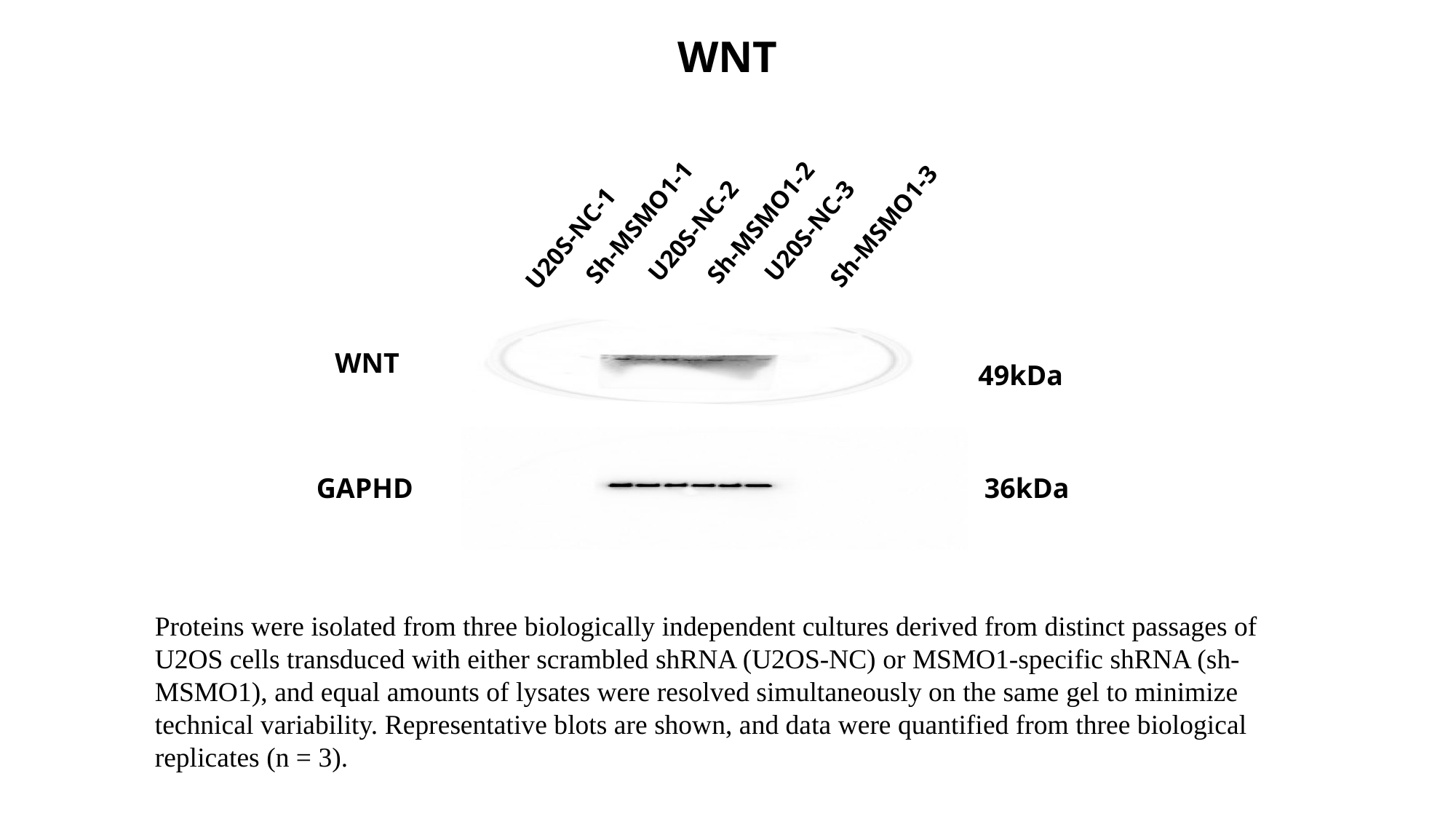

WNT
Sh-MSMO1-1
Sh-MSMO1-2
Sh-MSMO1-3
U20S-NC-2
U20S-NC-3
U20S-NC-1
WNT
49kDa
GAPHD
36kDa
Proteins were isolated from three biologically independent cultures derived from distinct passages of U2OS cells transduced with either scrambled shRNA (U2OS-NC) or MSMO1-specific shRNA (sh-MSMO1), and equal amounts of lysates were resolved simultaneously on the same gel to minimize technical variability. Representative blots are shown, and data were quantified from three biological replicates (n = 3).

## Slide 5
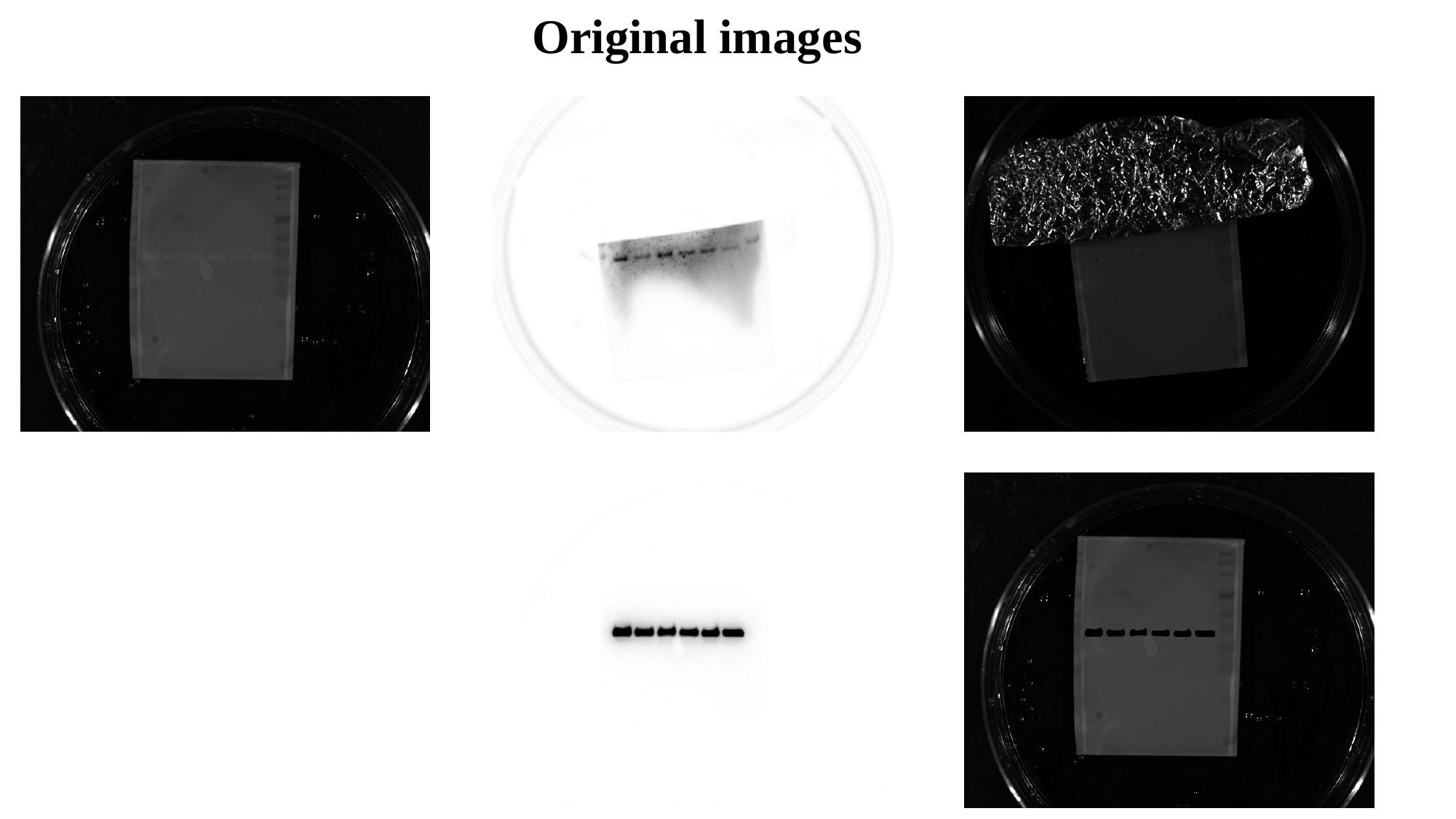

Original images

## Slide 6
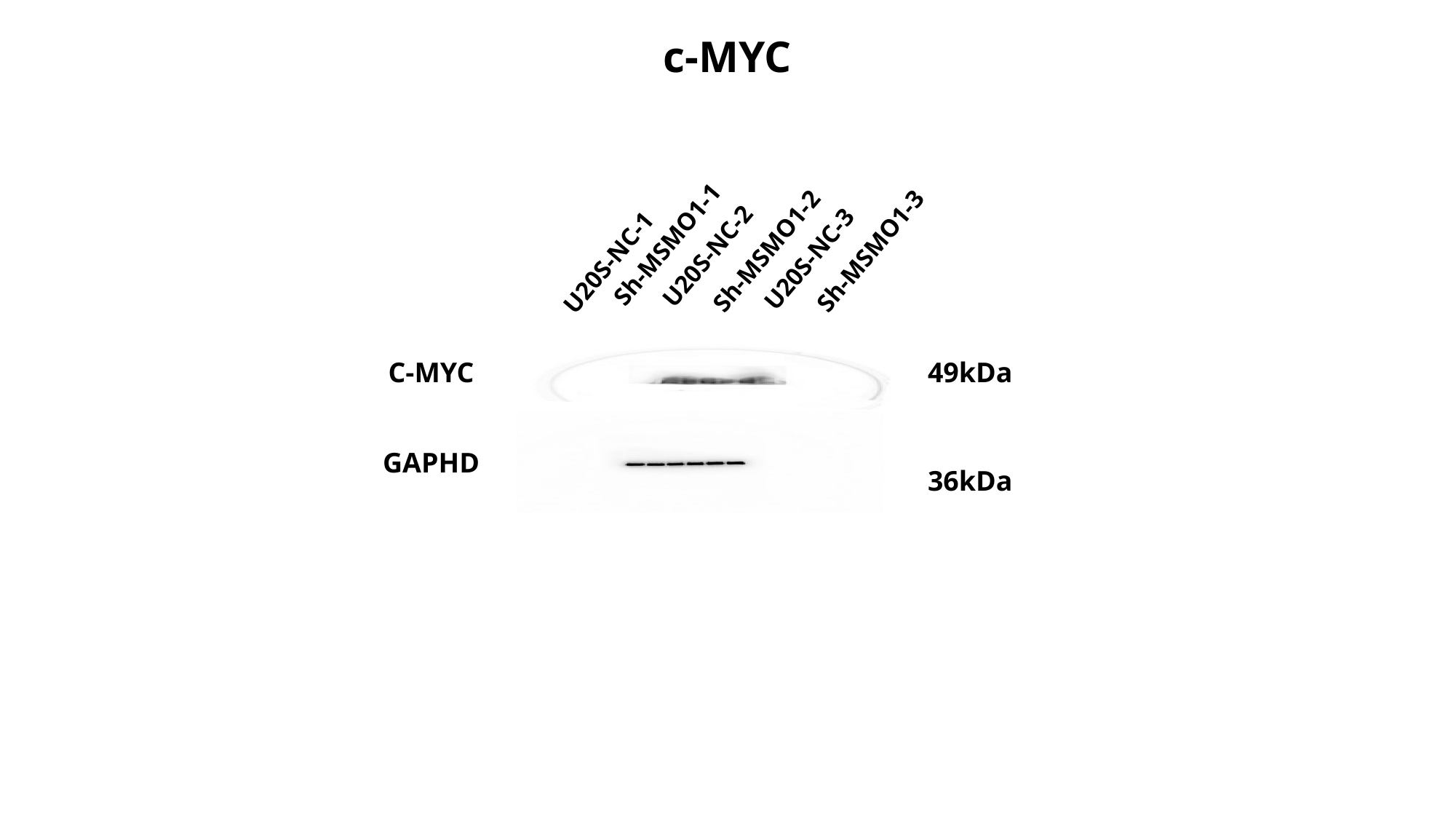

c-MYC
Sh-MSMO1-1
Sh-MSMO1-3
Sh-MSMO1-2
U20S-NC-2
U20S-NC-3
U20S-NC-1
C-MYC
49kDa
GAPHD
36kDa

## Slide 7
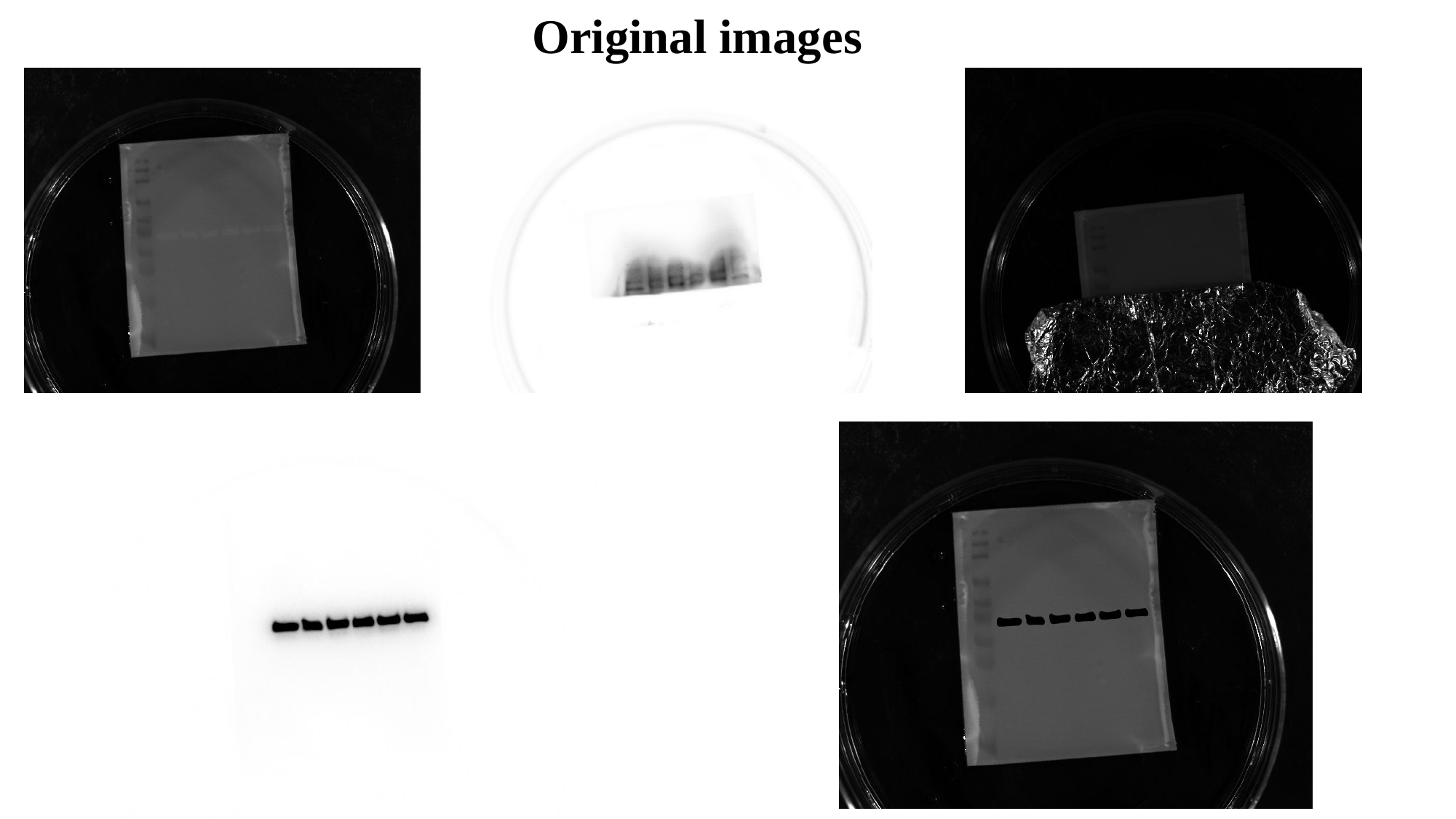

Original images

## Slide 8
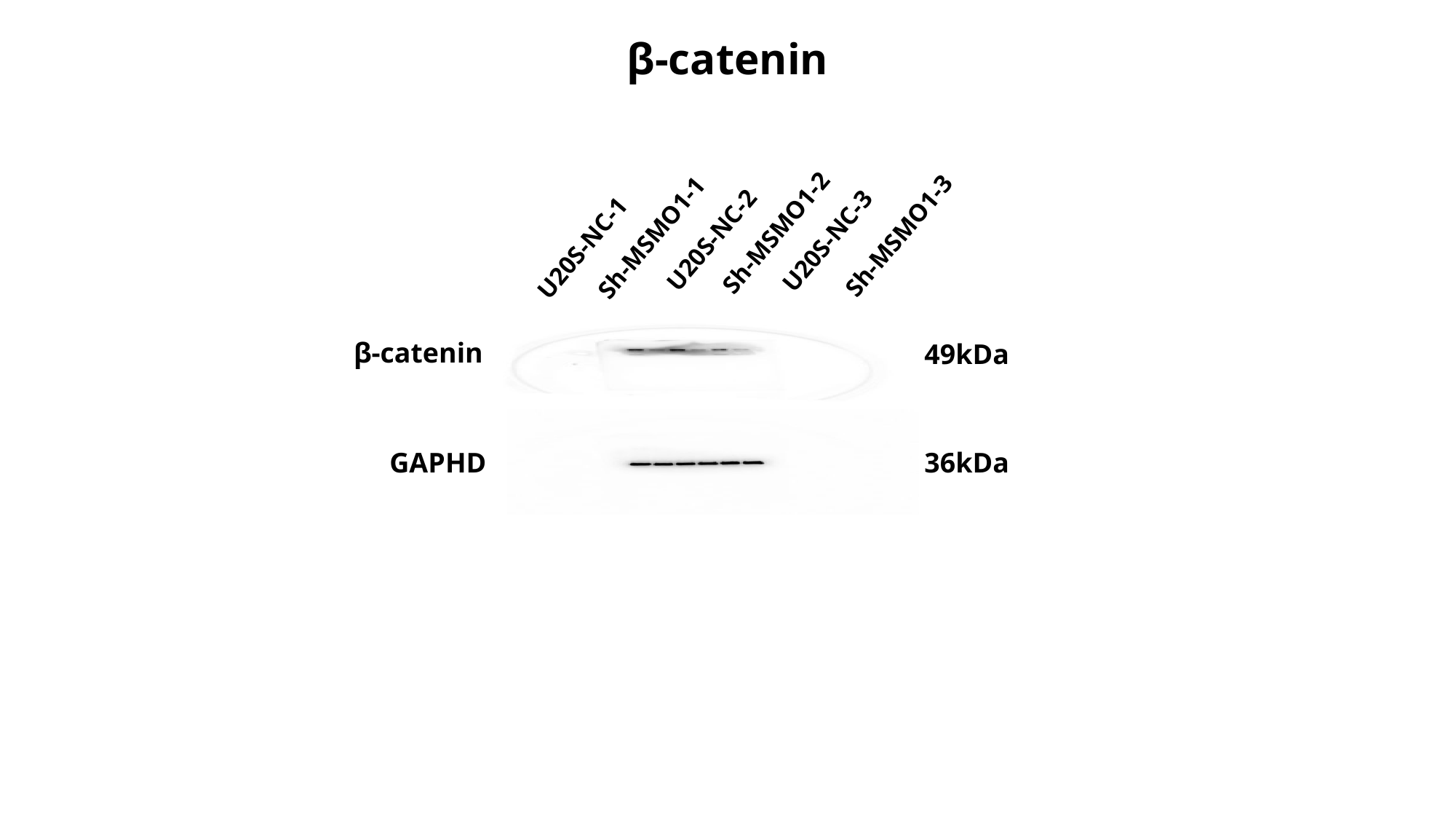

β-catenin
Sh-MSMO1-2
Sh-MSMO1-3
Sh-MSMO1-1
U20S-NC-2
U20S-NC-3
U20S-NC-1
β-catenin
49kDa
GAPHD
36kDa

## Slide 9
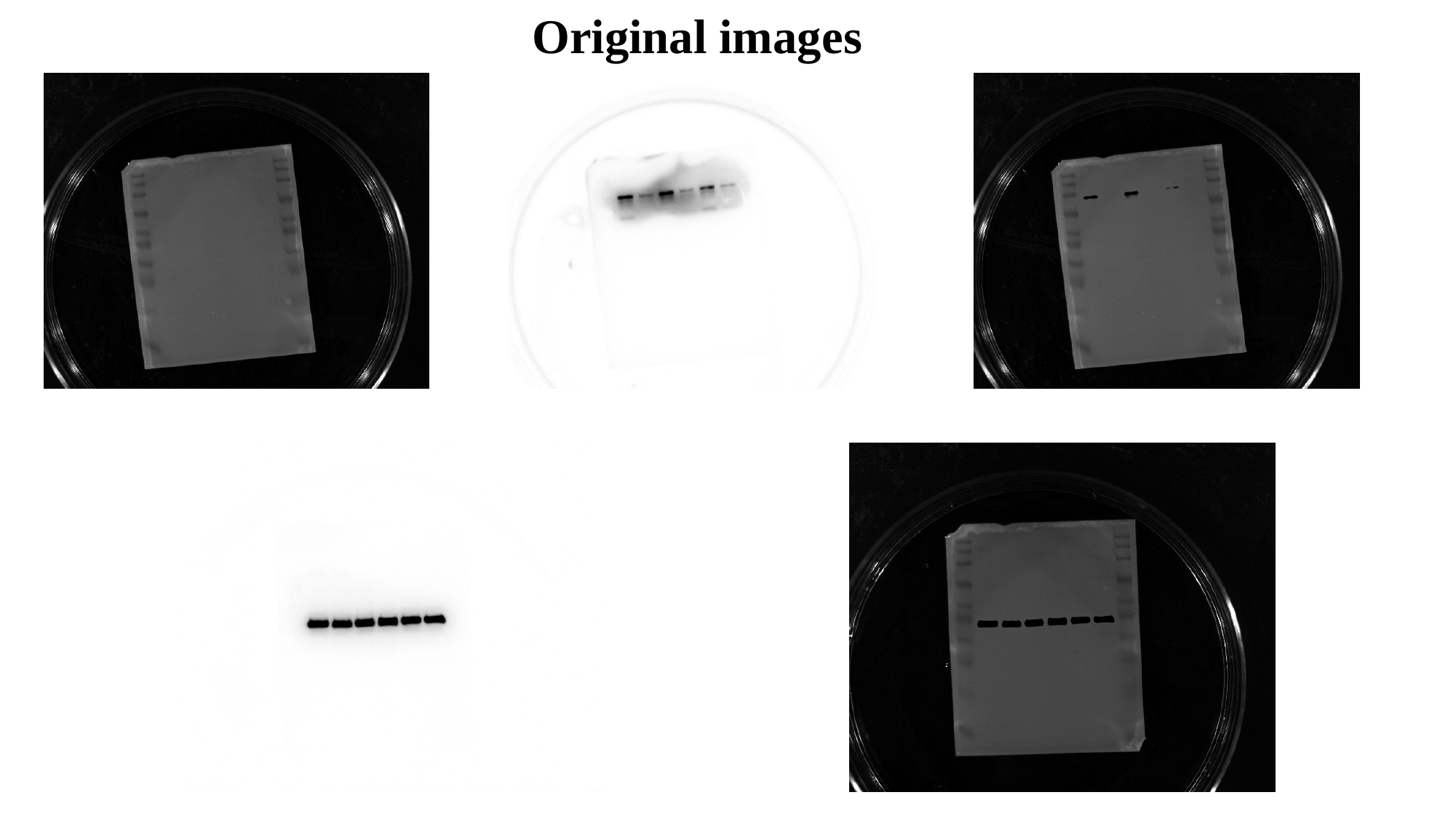

Original images

## Slide 10
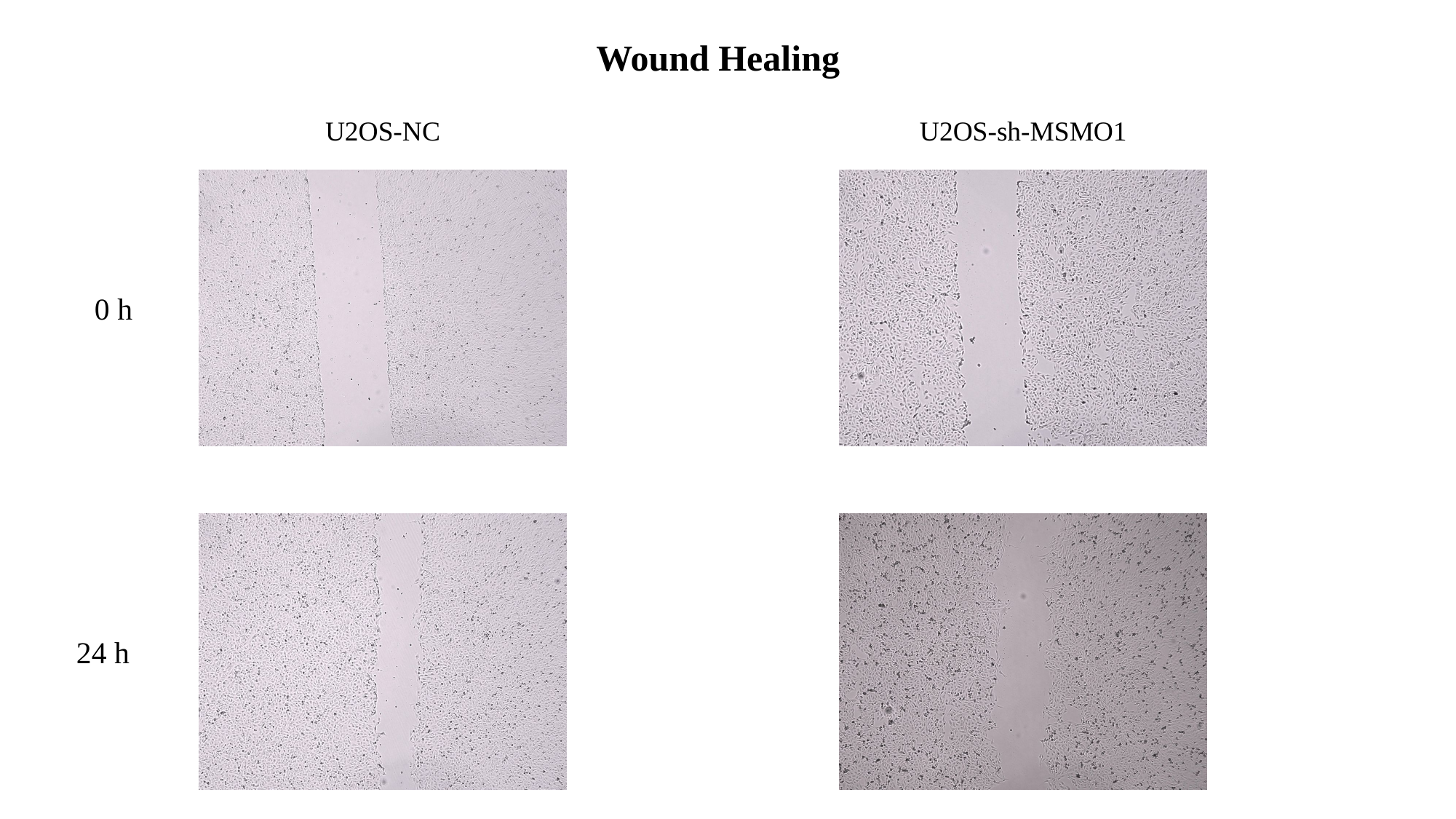

Wound Healing
U2OS-NC
U2OS-sh-MSMO1
0 h
24 h

## Slide 11
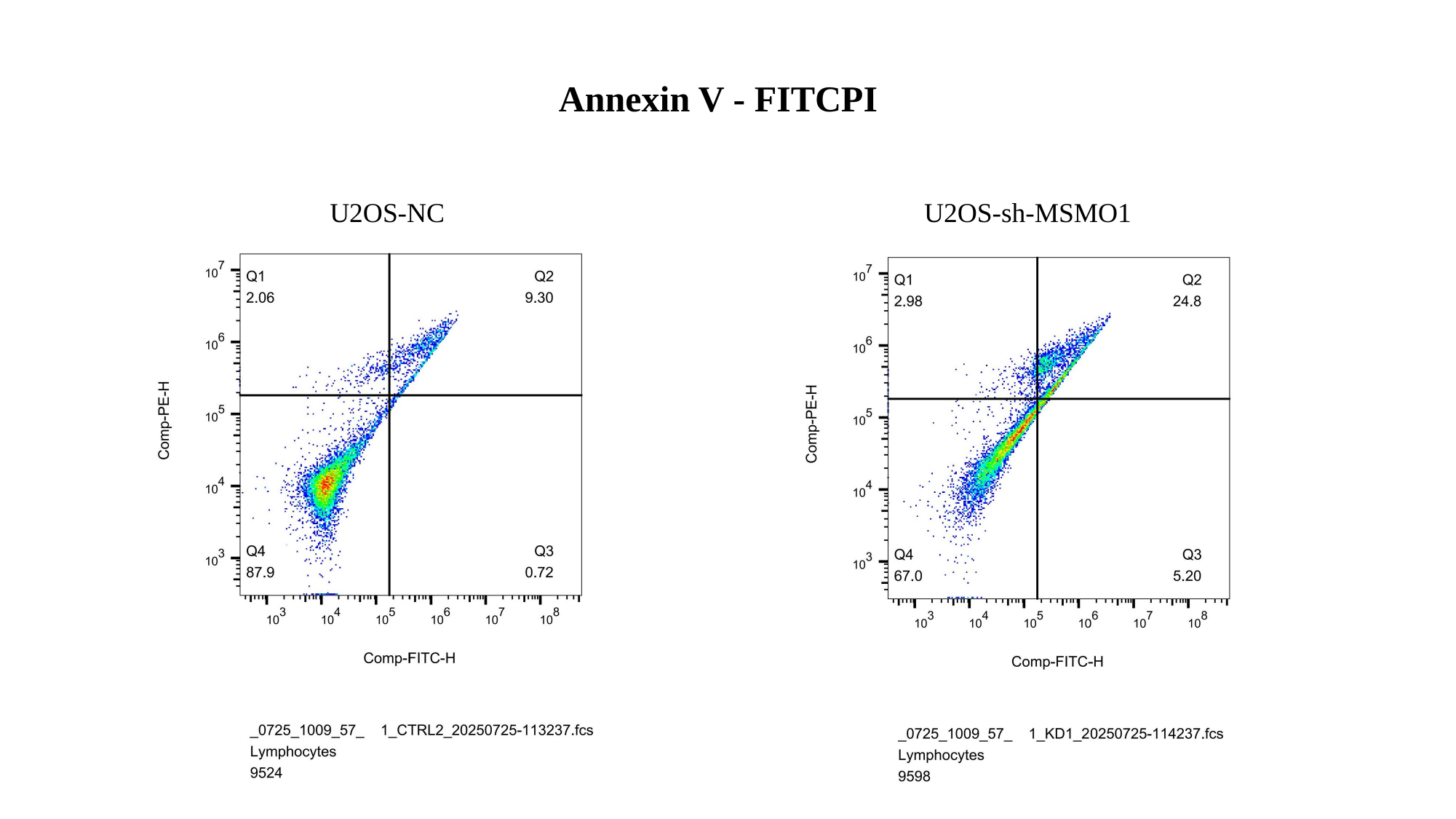

Annexin V - FITCPI
U2OS-NC
U2OS-sh-MSMO1

## Slide 12
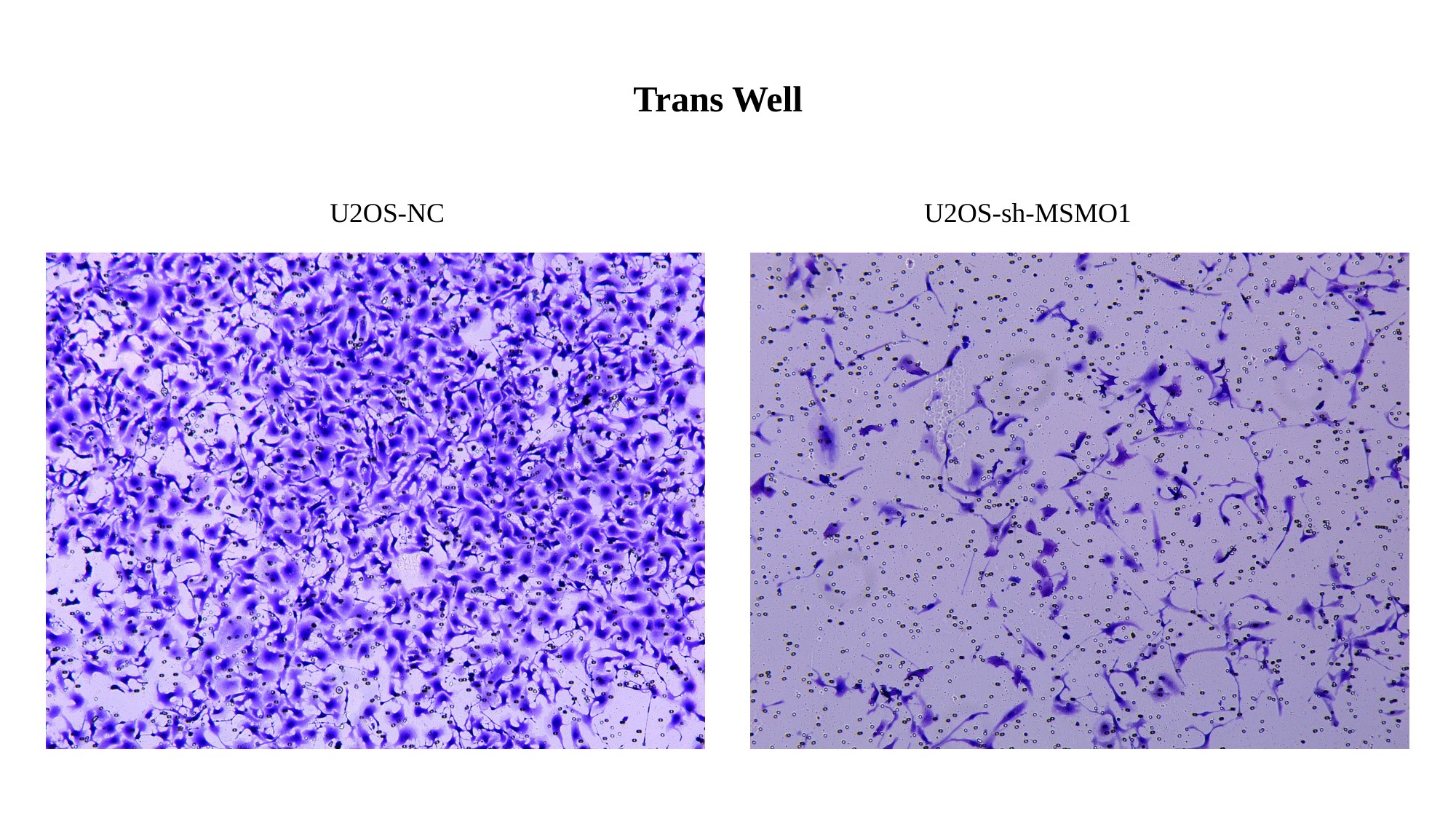

Trans Well
U2OS-NC
U2OS-sh-MSMO1
