# Supplementary material for: Integrative Single-Cell and Machine Learning Analysis Develops a Glutamine Metabolism–Based Prognostic Model and Identifies MSMO1 as a Therapeutic Target in Osteosarcoma
Source: Biomolecules. 2025 Nov 28;15(12):1664. doi: 10.3390/biom15121664 (PMC12731238; doi:10.3390/biom15121664)
Supplement: Supplementary file 1 [file biomolecules-15-01664-s001.zip › biomolecules-3977408-supplementary.pdf]

Supplementary Figures and Tables

Supplementary Table S1. The sequences of primers

Primers	Sequences (5′-3′)
H-GAPDH	F:5′-CTGGGCTACACTGAGCACC -3′<br>R:5′-AAGTGGTCGTTGAGGGCAATG -3′
SGMS2	F:5′-TCCTACGAACACTTATGCAAGAC-3′<br>R:5′-CCGGGTACTTTTTGGTGCCT-3′
GPX7	F:5′-CCCACCACTTTAACGTGCTC-3′<br>R:5′-GGCAAAGCTCTCAATCTCCTT-3′
CPE	F:5′-CATCTCCTTCGAGTACCACCG -3′<br>R:5′-CCGTGTAAATCCTGCTGATGG -3′
COL11A2	F:5′-GCTCCCCTCCTGACTCTCTAC-3′<br>R:5′-CCGGGTGACTCGCTTCTTG-3′
MSMO1	F:5′-TATGCTGGTTCTCGGCATCAT-3′<br>R:5′-CCAAAAATTCGATCCCACCATGT-3′

Supplementary Table S2. The sequences of primers for short hairpin (sh)-MSMO1

Primers	Sequences (5′-3′)
sh1-MSMO1	F:5′-<br>CCGGGCATAGACTCTTACACCACAACTCGAGTTGTGGT<br>GTAAGAGTCTATGCTTTTTG-3′<br>R:5′-<br>AATTCAAAAAGCATAGACTCTTACACCACAACTCGAG<br>TTGTGGTGTAAGAGTCTATGC-3′

sh2-MSMO1	F:5′-CCGGTGATTTGTGGAACCTATTATTCTCGAGAATAATAGGTTCCACAAATCATTTTTG-3′<br>R:5′-AATTCAAAAATGATTTGTGGAACCTATTATTCTCGAGAATAATAGGTTCCACAAATCA-3′
sh3-MSMO1	F:5′-CCGGCCTGAGAATCCTCTGCAAGAACTCGAGTTCTTGCAGAGGATTCTCAGGTTTTTG-3′<br>R:5′-AATTCAAAAACCTGAGAATCCTCTGCAAGAACTCGAGTTCTTGCAGAGGATTCTCAGG-3′

Supplementary Figures

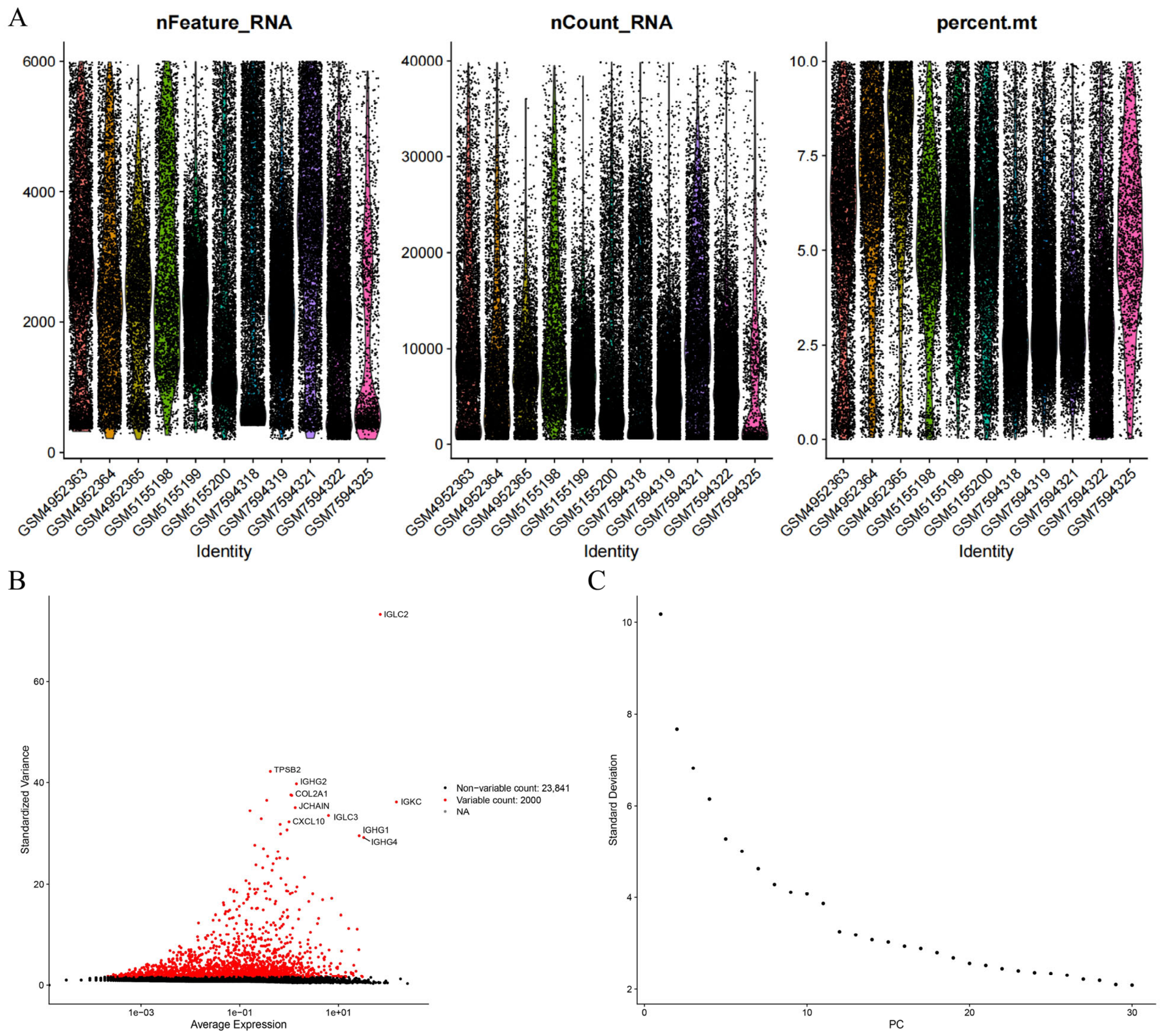

Supplementary Figure S1. Single-cell RNA sequencing (scRNA-seq) analysis. (A) The quality control for scRNA-seq data. (B) Top 2000 highly variable genes. (C) Top 30 principle components.

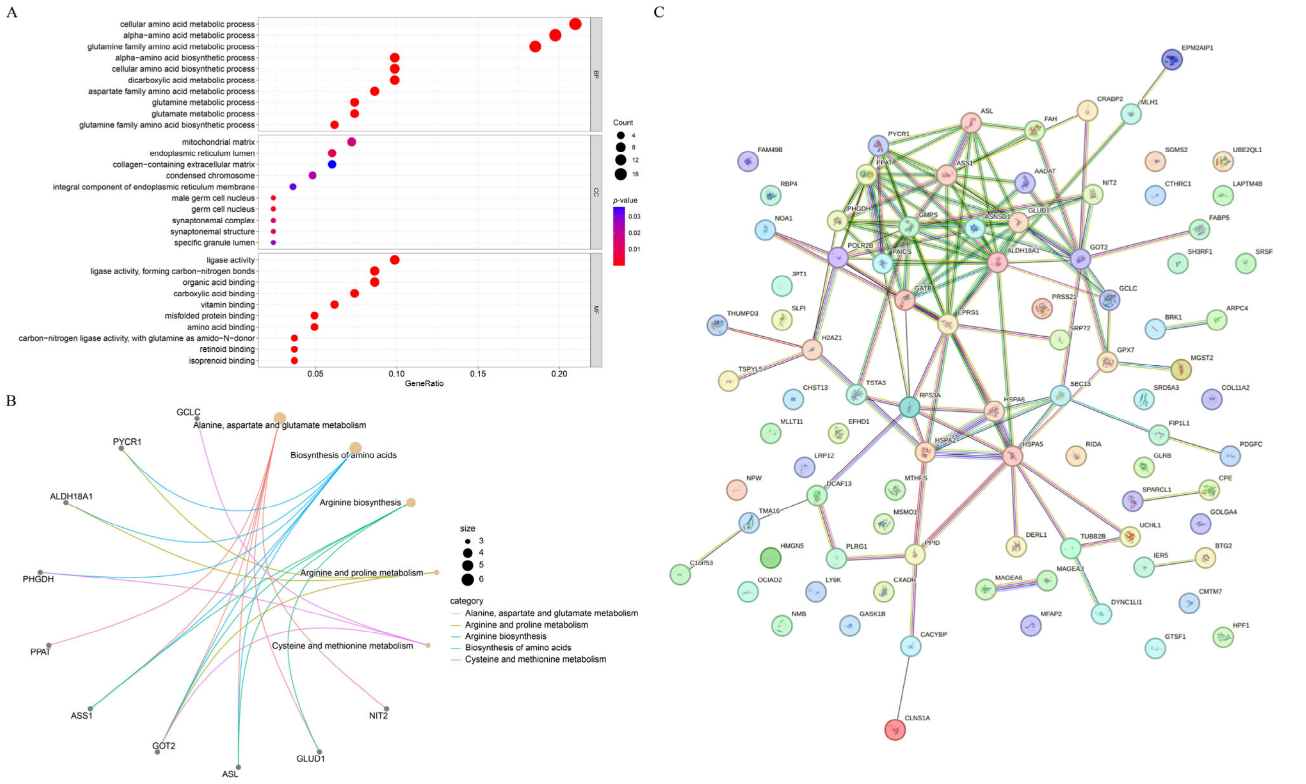

Supplementary Figure S2. Pathway enrichment analyses and Protein-protein interaction (PPI) network analysis of 91 upregulated differentially expressed genes (DEGs). (A-B) Gene Ontology (GO) (A) and Kyoto Encyclopedia of Genes and Genomes (KEGG). The gradient from red to blue indicates that the *p*-value is gradually increasing. (B) enrichment analyses for up-regulated DEGs. (C) PPI network of up-regulated DEGs.

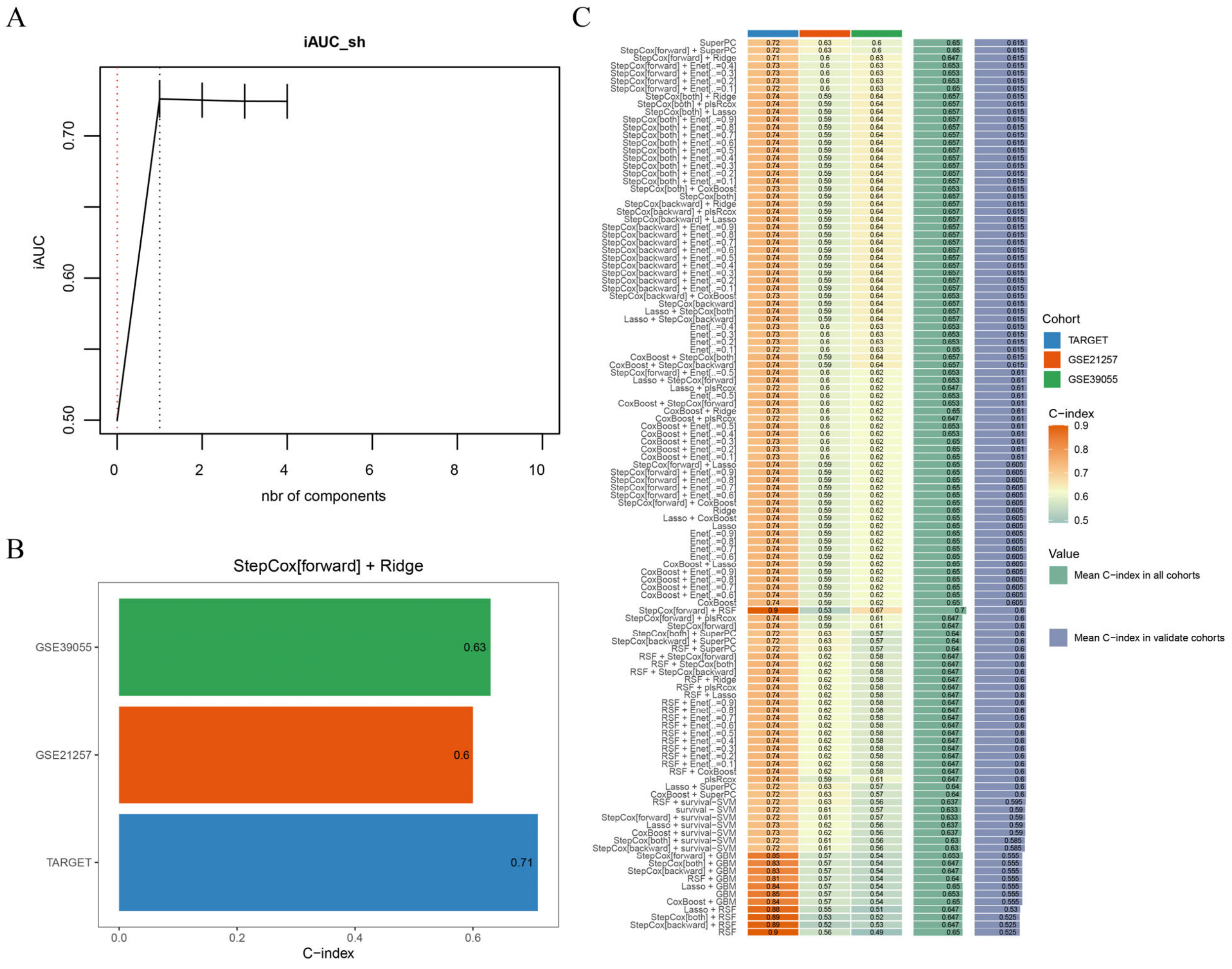

Supplementary Figure S3. Selection of the optimal model. (A) receiver operating characteristic curve (ROC) for machine learning algorithms. (B) C-index value in each dataset. Different colors represent different datasets. (C) The results of 101 machine learning algorithm combinations.

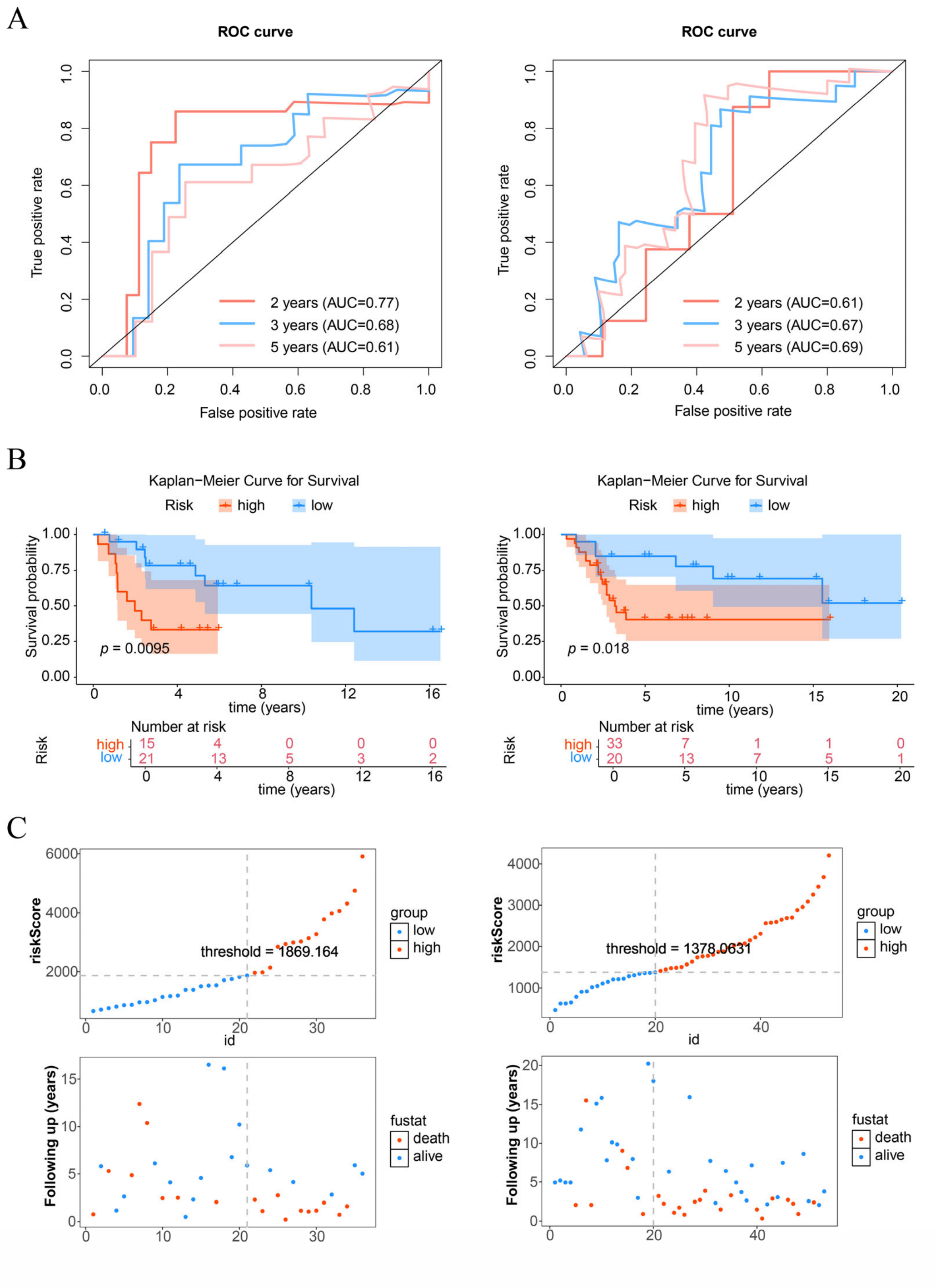

Supplementary Figure S4. Validation of the prognostic model. (A) ROC curve in validation sets (GSE39055, GSE21257). Curves of different colors represent different years. (B) Kaplan–Meier (KM) curve in validation sets. The red curve represents the high-risk group, and the

blue curve represents the low-risk group. (C) Survival curve in validation sets. Blue denotes alive samples; red denotes death samples.

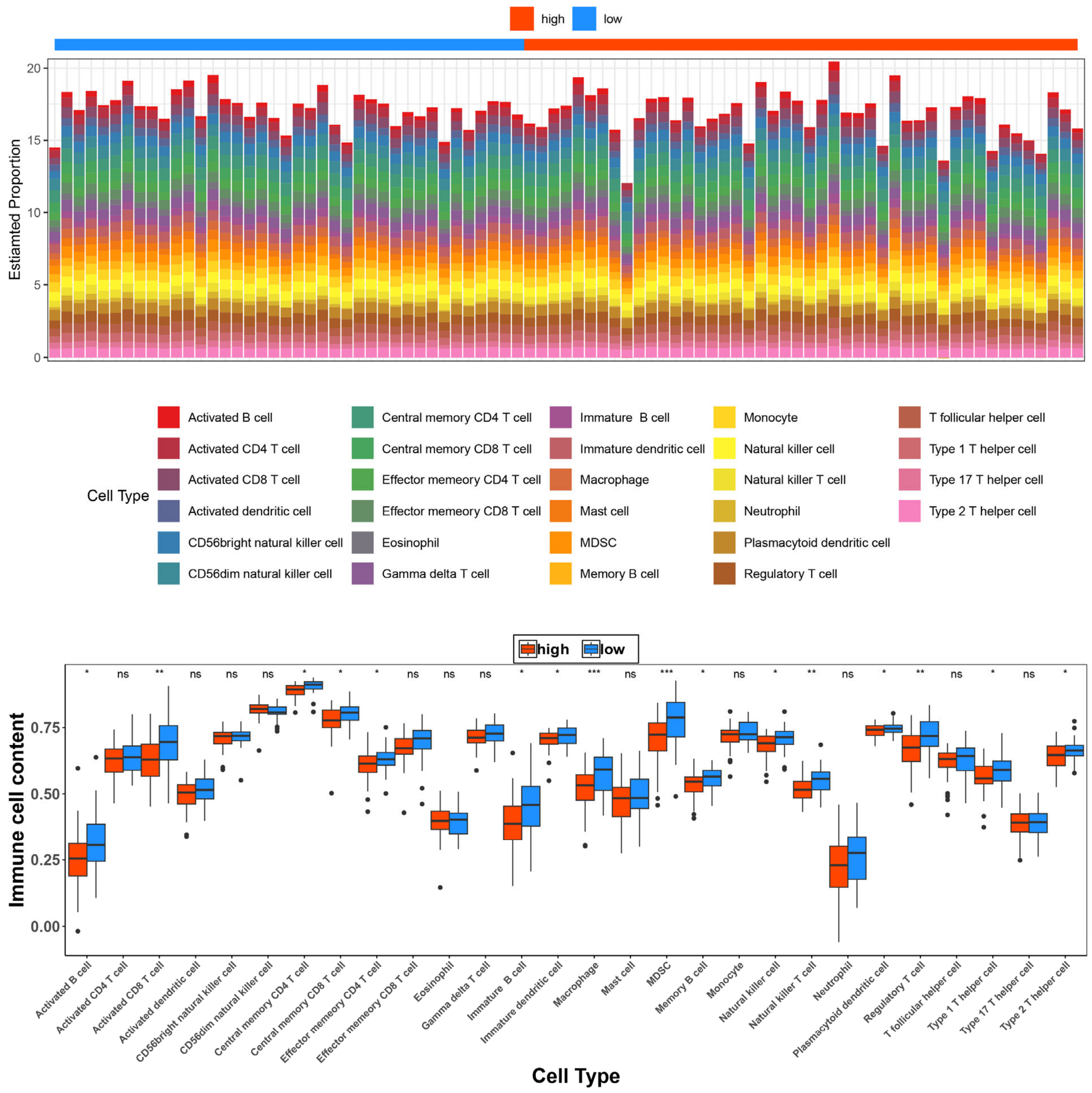

Supplementary Figure S5. Employing the "GSVA" package to evaluate differences in immune cell infiltration levels between high- and low-risk groups of osteosarcomas. ns=no significance. The red represents the high-risk group, and the blue represents the low-risk group. * $p < 0.05$, ** $p < 0.01$, *** $p < 0.001$.

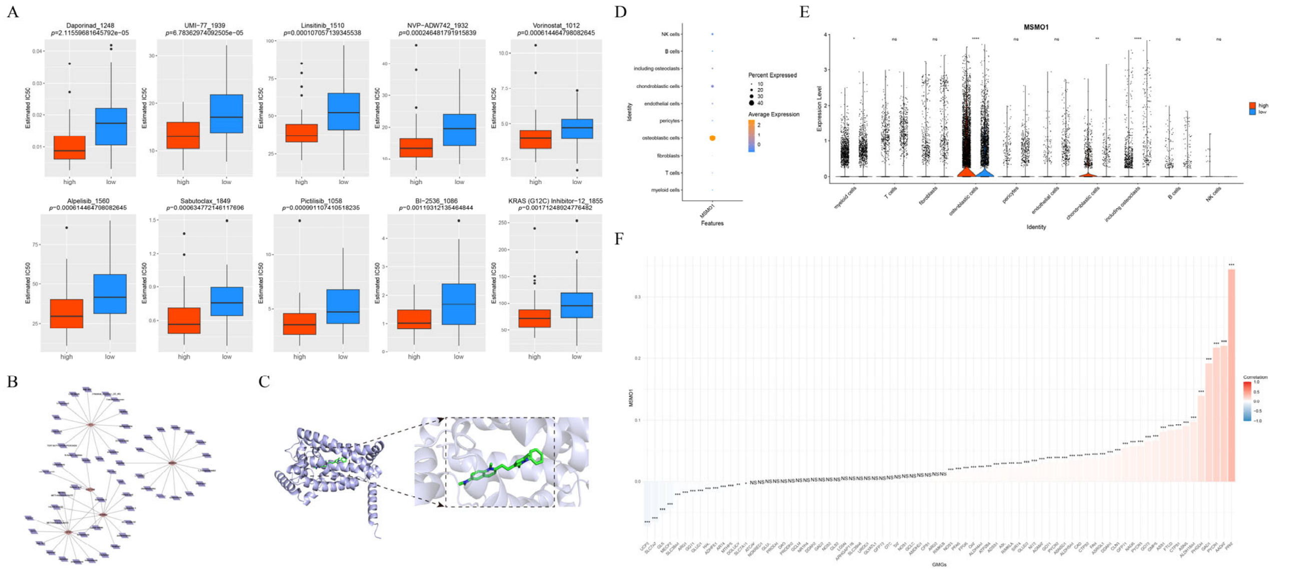

Supplementary Figure S6. Potential targeted therapy for OS. (A) The difference of drug sensitivity between high and low risk groups. The red represents the high-risk group, and the blue represents the low-risk group. (B) A mRNA-drug regulatory network. (C) The molecular docking of MSMO1 and pyrvinium. (D) The expression of MSMO1 in cell subtypes. (E) The difference of MSMO1 expression in cell subtypes between high and low risk groups. (F) The correlation between MSMO1 with GRGs.

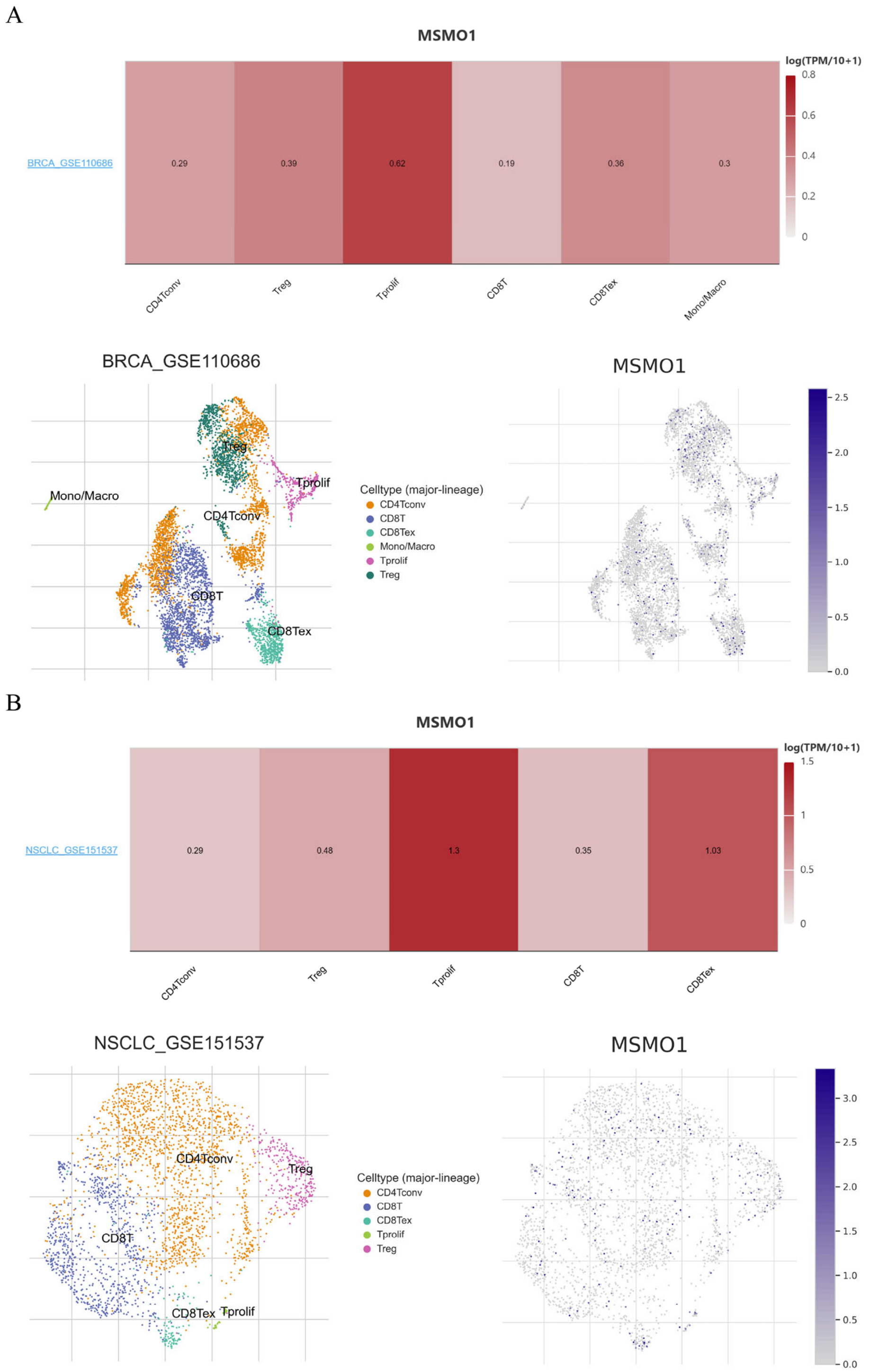

Supplementary Figure S7. The expression levels of MSMO1 in different immune cells of breast cancer (A) and non-small cell lung cancer (B). Darker colours indicate higher levels of gene expression in the heatmap.
